# Distinct hybridization modes in wide- and narrow-ranged lineages of *Causonis* (Vitaceae)

**DOI:** 10.1186/s12915-023-01718-8

**Published:** 2023-10-09

**Authors:** Jinren Yu, Hong Zhao, Yanting Niu, Yichen You, Russell L. Barrett, Rindra Manasoa Ranaivoson, Romer Narindra Rabarijaona, Gaurav Parmar, Langxing Yuan, Xiaofeng Jin, Pan Li, Jianhua Li, Jun Wen, Zhiduan Chen, Limin Lu

**Affiliations:** 1 State Key Laboratory of Plant Diversity and Specialty Crops, Institute of Botany, Chinese Academy of Sciences, Beijing, 100093 China; 2 China National Botanical Garden, Beijing, 100093 China; 3 University of Chinese Academy of Sciences, Beijing, 100049 China; 4 School of Life Science, Shanxi Normal University, Taiyuan, 030031 China; 5 National Herbarium of New South Wales, Australian Botanic Garden, Locked Bag 6002, Mount Annan, NSW 2567 Australia; 6 School of Biological, Earth and Environmental Sciences, The University of New South Wales Sydney, Kensington, NSW 2052 Australia; 7 National Botanical Garden, Godawari, 44709 Nepal; 8 Tropical Crops Genetic Resources Institute, Chinese Academy of Tropical Agricultural Sciences, Haikou, 571101 China; 9 Zhejiang Provincial Key Laboratory of Forest Aromatic Plants-Based Healthcare Functions/School of Forestry and Bio-Technology, Zhejiang A&F University, Hangzhou, 311300 China; 10 Laboratory of Systematic & Evolutionary Botany and Biodiversity, College of Life Sciences, Zhejiang University, Hangzhou, 310058 China; 11 Biology Department, Hope College, Holland, MI 49423 USA; 12 Department of Botany, National Museum of Natural History, MRC166, Smithsonian Institution, Washington, DC, 20013-7012 USA; 13 Sino-Africa Joint Research Center, Chinese Academy of Sciences, Wuhan, 430074 China

**Keywords:** Distribution range, *Causonis*, Hybridization, Whole genome duplication, Allopolyploidization, Reticulate evolution

## Abstract

**Background:**

Explaining contrasting patterns of distribution between related species is crucial for understanding the dynamics of biodiversity. Despite instances where hybridization and whole genome duplication (WGD) can yield detrimental outcomes, a role in facilitating the expansion of distribution range has been proposed. The Vitaceae genus *Causonis* exhibits great variations in species’ distribution ranges, with most species in the derived lineages having a much wider range than those in the early-diverged lineages. Hybridization and WGD events have been suggested to occur in *Causonis* based on evidence of phylogenetic discordance. The genus, therefore, provides us with an opportunity to for explore different hybridization and polyploidization modes in lineages with contrasting species’ distribution ranges. However, the evolutionary history of *Causonis* incorporating potential hybridization and WGD events remains to be explored.

**Results:**

With plastid and nuclear data from dense sampling, this study resolved the phylogenetic relationships within *Causonis* and revealed significant cyto-nuclear discordance. Nuclear gene tree conflicts were detected across the genus, especially in the japonica-corniculata clade, which were mainly attributed to gene flow. This study also inferred the allopolyploid origin of the core *Causonis* species, which promoted the accumulation of stress-related genes. *Causonis* was estimated to have originated in continental Asia in the early Eocene, and experienced glaciation in the early Oligocene, shortly after the divergence of the early-divergent lineages. The japonica-corniculata clade mainly diversified in the Miocene, followed by temperature declines that may have facilitated secondary contact. Species distribution modeling based on current climate change predicted that the widespread *C. japonica* tends to be more invasive, while the endemic *C. ciliifera* may be at risk of extinction.

**Conclusions:**

This study presents *Causonis*, a genus with complex reticulate evolutionary history, as a model of how hybridization and WGD modes differ in lineages of contrasting species’ geographic ranges. It is important to consider specific evolutionary histories and genetic properties of the focal species within conservation strategies.

**Supplementary Information:**

The online version contains supplementary material available at 10.1186/s12915-023-01718-8.

## Background

The geographic distribution ranges of species may vary dramatically, even among clades with close relationships [1–5]. Indeed, in some cases, geographic barriers constitute the boundaries of a species’ distribution range, yet commonly, the limit is regarded as a spatial expression of ecological niche [6]. The boundary of species distributions seems to be partially set by the ability of edge populations to colonize new environments beyond their current range [1, 4, 5, 7]. What is happening at the edge of a species’ range resembles a dynamic race: the distribution range expands when edge populations successfully adapt to new environments before facing extinction; conversely, ranges contract when adaptation fails [3, 7, 8]. During this process, the adaptability of edge populations may be constrained by gene flow from the larger core population [3] and the accumulation of deleterious mutations [9]. Therefore, to some extent, accelerating the generation of adaptive innovations, as well as delaying the extinction of edge populations, can facilitate distribution range expansion [2, 8, 10, 11].

During the natural range expansion and contraction of plants, both hybridization and whole genome duplication (WGD) are believed to have significant roles when present [12–14]. Although hybridization can have detrimental effects on fitness, numerous studies have proposed that it can also serve as a creative force in evolution by generating novel phenotypes that enable species to colonize new habitats. This may occur through the transfer of adaptive alleles or genetic recombination across species [5, 13–16]. Moreover, hybridization can help maintain or augment the population size of small edge populations by generating transient fitness through heterosis, thus preventing their extinction before adaptation [5, 17]. Interspecific hybridization, by enhancing biological functions and generating genetic diversity, may also contribute to the rapid colonization of invasive plants [18, 19]. For plants that have experienced WGD, the rewiring of biological pathways resulting from duplicated gene families can trigger sudden mutations across generations, facilitating adaptation to abrupt environmental changes. This, in turn, increases the survival rate in the population of the new colony [20–23]. The redundant gene copies and heightened genetic diversity resulting from WGD also provide plants with more chances to acquire evolutionary innovations [24, 25]. While numerous previous studies have suggested that WGD may facilitate distribution range expansion [26–28], this phenomenon deserves further investigation utilizing strategies that bridge genomics and biogeography, such as phylogenetic network inference, WGD identification, biogeographic reconstruction, and species distribution modeling.

*Causonis* Raf., a recently resurrected genus in the grape family (Vitaceae), is widely distributed in tropical, subtropical, and temperate regions from Asia to Australia, including the Pacific islands [29, 30]. It currently includes 16 species and four varieties, and species in different lineages show great variations in distribution range (Additional file 1: Table S1) [30, 31]. Most species from the early-diverged lineages of the genus are confined to a particular region [30]. For instance, *Causonis ciliifera* is only found in Hainan, China, and the coastal areas of Vietnam, while *C. timoriensis* var. *mekongensis* is endemic to Yunnan, China [30]. In contrast, species within the derived lineages are widespread. For example, *C. japonica* has a broad distribution range extending almost throughout the entire monsoon region in East and South Asia [30, 32]. *Causonis japonica* has been recognized as an invasive plant in eastern North America and is well known as “bushkiller” for damaging native vegetation by overtopping local plants [33, 34].

Potential hybridization or WGD events have been suggested to occur in *Causonis*, which might be associated with the wide distributions in some lineages [30, 31]. Previous research has suggested cyto-nuclear discordances across the phylogeny of *Causonis* [30], which was inferred to be a result of introgressive plastid capture [35, 36]. Moreover, polyploids have been frequently reported in the widespread species *C. japonica* and *C. trifolia* (2n = 4x = 40 and 2n = 6x = 60) [37–39]. This pattern may imply plausible connections between polyploidization events and the expansion of distribution ranges. The variability in distribution ranges coupled with diverse hybridization or polyploidization histories across *Causonis* species offers an opportunity to investigate the contributing factors to distribution range variation from both the genetic and biogeographic aspects.

To test the respective contributions of hybridization and polyploidization in affecting distribution ranges of *Causonis* species in a framework of biogeographic history, it is necessary to clarify phylogenetic relationships. However, phylogenetic relationships within *Causonis* have not been completely resolved. Although previous studies have explored relationships among main clades of the genus using multiple plastid and nuclear loci [30, 31, 40], species within *Causonis* were not fully sampled and phylogenetic positions of some species were not strongly supported. Phylogenetic uncertainty within *Causonis* may have been partially caused by potential hybridization or polyploidization events leading to discordant phylogenetic signals that blur our understanding of the true ancient divergences in evolutionary history [41–43]. From this arises the major challenge in the field of systematic and evolutionary biology: the identification of the sources of gene tree conflicts, including not only hybridization and/or WGD events but also other processes such as incomplete lineage sorting (ILS) and errors in gene tree estimation. Despite the difficulties, determining the phylogenetic placements of hybridization and polyploidization events is a prerequisite to inferring the roles they played in shaping distribution ranges of species throughout the course of biogeographic evolutionary history.

This study aims to resolve phylogenetic relationships within *Causonis* using carefully filtered single-copy orthologs and plastome protein-coding sequences based on dense taxon sampling. With a robust phylogeny obtained, the causes of gene tree conflicts and cyto-nuclear discordance between species trees were explored, focusing on the role of hybridization, ILS, and gene tree estimation error. To identify hybridization events, the level of gene flow was assessed and their locations on the phylogeny were determined based on reticulate index,* D*-statistic test, and phylogenetic network inference. This study also inferred potential ancient polyploidization events in *Causonis* based on transcriptome data. Finally, the past and future distribution changes of widely versus narrowly distributed species in the context of global climate change were estimated and compared. This study illustrates the different hybridization and polyploidization histories of *Causonis* species characterized by wide or narrow distributions, all within the framework of biogeography. We explored the conservation implications and suggest that the genomic and biogeographic evolutionary history of the focal species should be considered in conservation planning and actions.

## Results

### Phylogenetic relationships within *Causonis*, cyto-nuclear discordance and nuclear gene tree conflicts

To resolve the phylogenetic relationships within *Causonis*, we sampled 82 individuals representing 16 species and three varieties of *Causonis*, which is the most comprehensive sampling of *Causonis* to date, and gathered whole genome sequencing data (Additional file 1: Table S2). Six species of *Pseudocayratia* and 11 species of *Tetrastigma* were included as outgroups, as the two genera have been identified as the closest relatives of *Causonis* [30] (Additional file 1: Table S3). Among these sampled individuals, transcriptomes of six species were sequenced, including five species of *Causonis* (*C. ciliifera*, *C. daliensis*, *C. japonica* var. *pseudotrifolia*, *C. timoriensis* var. *mekongensis*, and *C. trifolia*) and one species of *Pseudocayratia* (*P. speciosa*).

After quality trimming of the raw reads and removing the organelle reads, the total number of clean reads generated from RNA sequencing of each species ranged from 19,441,421 to 55,618,420, with an average of 34,025,426 reads per species (Additional file 1: Table S4). Transcriptomes were assembled with an average BUSCO completeness of 85.7% (Additional file 2: Fig. S1), and the transcripts were well mapped by the raw reads (Additional file 1: Table S5). After filtering out genes showing potential long-branch attraction and saturation from identified “single-copy orthologs”, and further pruning for homologs with monophyletic and single-copy outgroup taxa, we included 810 orthologs in the phylogenetic reconstruction. The number of parsimony-informative sites in the trimmed gene alignments ranged from 49 to 3632, with an average number of 628 (Additional file 1: Table S6). We also extracted 79 plastid genes from the whole genome sequencing (WGS) data to explore cyto-nuclear discordance in *Causonis*.

**Fig. 1:**
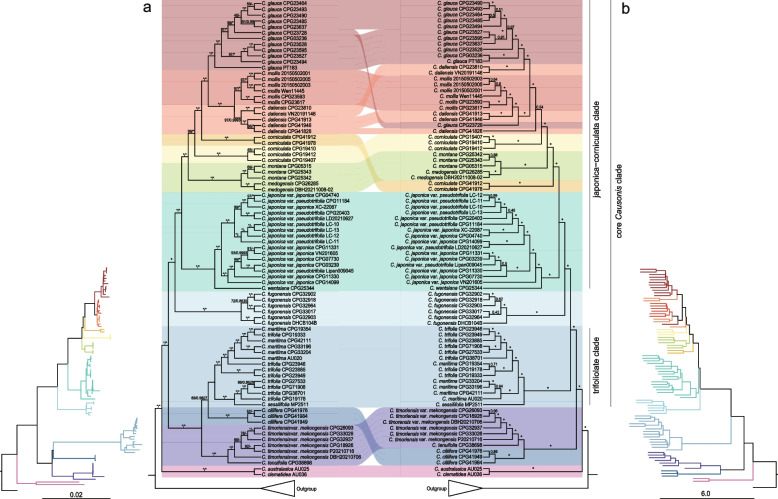
Cyto-nuclear discordance in *Causonis* phylogenies. **a** Phylogenies of *Causonis* based on the 136taxa-79pd dataset using concatenated approach, including ML and BI methods. To the left is the phylogram. **b** Phylogenies of *Causonis* constructed with the 136taxa-810nu dataset using MSC method. To the right is the phylogram. Numbers above branches in **a** show the ML BS/BI PP values and those in **b** are local posterior probability support values. “*” denotes the maximal support. The main clades of *Causonis* are marked in different colors

Phylogenetic trees were reconstructed using the concatenation-based approaches including maximum likelihood (ML) method and Bayesian inference (BI), and the coalescent-based approach, multispecies coalescent (MSC) method, with a nuclear dataset (136taxa-810nu) and a plastid dataset (136taxa-79pd) (Additional file 1: Table S7). As the MSC topologies using gene trees respectively collapsing nodes with bootstrap support (BS) values below 10, 40, and 70% were almost the same, the MSC tree with a BS cut-off of 10% was selected for further discussion, following Zhang et al. [44]. All the retrieved trees had an overall topology concordant with Parmar et al. [30] and highly supported the monophyly of most species of *Causonis* (including the taxonomically redefined *C. japonica*), except that *C. maritima* and *C. trifolia* had individuals nested within each other, and the individuals of *C. corniculata* were divided into two clades (*C. corniculata* I: CPG41912 and CPG41978, *C. corniculata* II: CPG19407, CPG19410, and CPG19412) (Fig. 1 and Additional file 2: Figs. S2 and S3). For the 136taxa-79pd dataset, the main difference in phylogenies obtained from the concatenated-based and coalescent-based methods was the phylogenetic position of *C. ciliifera*. In the former approaches, *C. ciliifera* was sister to the trifoliolate clade, whereas in the latter *C. ciliifera* was clustered with the clade consisting of *C. timoriensis* var. *mekongensis* and *C. tenuifolia*, but both topologies received low support (ML BS = 69%, BI PP = 0.9607, and 0.43 in the MSC tree; Fig. 1a and Additional file 2: Fig. S3). For the 136taxa-810nu dataset, the MSC method produced trees similar to those generated by the concatenation-based approach (Fig. 1b and Additional file 2: Figs. S2 and S3).

According to the 136taxa-810nu dataset, two species endemic to Australia, *C. clematidea* and *C. australasica*, formed the clade sister to the remaining species of *Causonis*. *Causonis ciliifera* was sister to the clade consisting of *C. timoriensis* var. *mekongensis* and *C. tenuifolia*. *Causonis maritima* and *C. trifolia* formed a clade with exclusively trifoliolate leaves, but which was sister to *C. sessilifolia* with pedately 5-foliolate leaves. *Causonis fugongensis* was highly supported as monophyletic, and so was *C. japonica* including two varieties, *C. japonica* var. *japonica* and *C. japonica* var. *pseudotrifolia*, which formed a clade with *C. wentsiana*. *Causonis montana* and *C. medogensis* formed a subclade, and individuals of *C. glauca*, *C. daliensis*, and *C. mollis* formed another subclade. The above two subclades and two separate lineages of *C. corniculata* formed a major clade (Fig. 1b and Additional file 2: Fig. S2). This major clade, combined with the *C. japonica* clade, was defined as the “japonica-corniculata clade”.

The phylogenies of *Causonis* showed significant cyto-nuclear discordance. Relationships among subclades in the japonica-corniculata clade based on the 136taxa-79pd dataset varied greatly from those based on the nuclear genes. The plastid data better supported the monophyly of each species in the japonica-corniculata clade, except that *C. corniculata* was resolved as a grade including two successive lineages (Fig. 1a). Although the 136taxa-79pd dataset did not fully resolve the deep-branching relationships of *C. ciliifera*, *C. timoriensis* var. *mekongensis*, *C. tenuifolia*, and the trifoliolate clade, it strongly supported these clades clustered together. However, the 136taxa-810nu dataset showed the trifoliolate clade sister to a clade including the japonica-corniculata clade and the *C. fugongensis* clade; the combination of these three clades was herein defined as the “core *Causonis* clade” (Fig. 1 and Additional file 2: Fig. S3). Extensive cyto-nuclear discordance and low support values indicated that the MSC method, which accommodates ILS during species tree inference, cannot fully resolve phylogenetic relationships within *Causonis*, suggesting that other biological processes (e.g., hybridization) may also have played significant roles in causing conflicts.

**Fig. 2:**
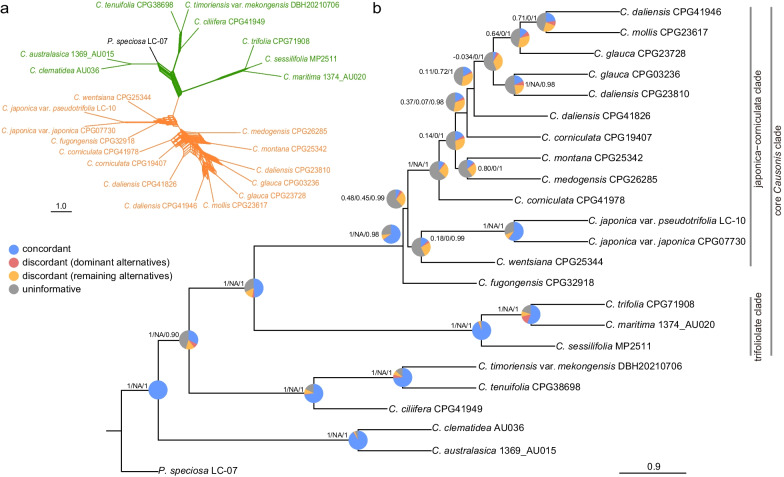
Topological conflicts of gene trees in *Causonis* based on the 23taxa-810nu dataset. **a** Supernetwork constructed with SplitsTree based on 810 rooted ML gene trees (10% majority-rule consensus trees), where parallelograms indicate incongruences among gene trees. Branches in orange indicate the japonica-corniculata clade, green indicates other clades in *Causonis*, and black represents the outgroup. **b** MSC tree constructed with ML gene trees. The pie chart at each node represents the proportion of genes supporting congruent relationships (blue), the dominant alternative (red), the remaining conflicting alternatives (yellow), and genes with less than 75% BS value for that node (gray). The numbers beside the pie chart denote quartet scores (quartet concordance/quartet differential/quartet informativeness)

The nuclear gene tree conflicts were then assessed to investigate the cause of gene tree conflicts in the phylogenetic relationships within *Causonis* using a subset of samples in the nuclear dataset (23taxa-810nu, see Additional file 1: Table S7). As visualized by SplitsTree, the nuclear gene tree conflicts were mostly concentrated in the japonica-corniculata clade and deep nodes of early-diverged lineages (Fig. 2a). Both the internode certainty all (ICA) value and quartet concordance score indicated that almost all nodes in the core *Causonis* clade showed high levels of discordance (Fig. 2b). Although a large proportion of gene trees had low support for nodes within the core *Causonis* clade, the quartet informativeness score showed that most of the gene alignments were phylogenetically informative (Fig. 2b).

### Widespread hybridizations across the phylogeny of *Causonis*

**Fig. 3:**
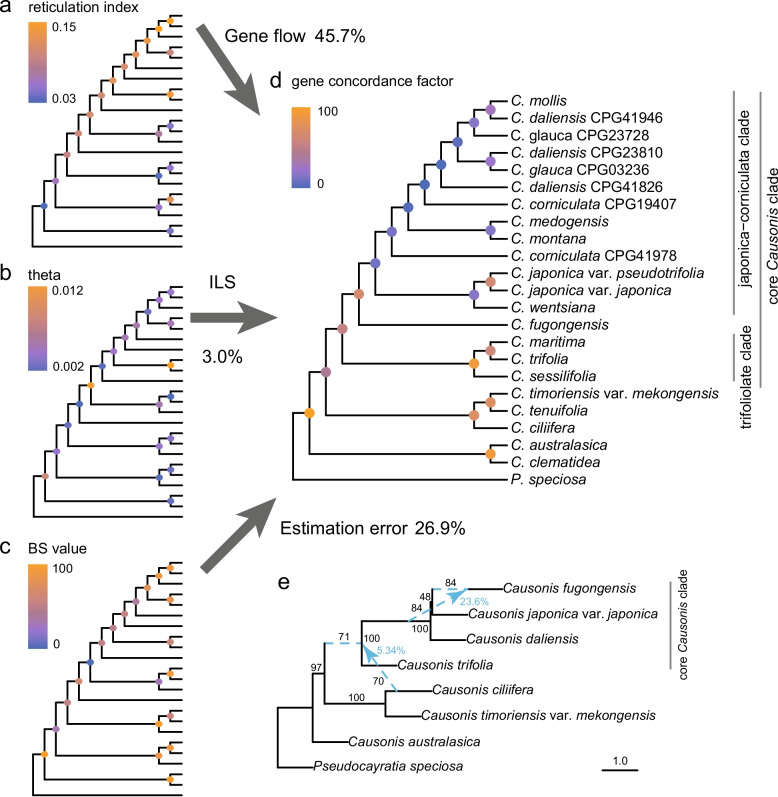
Detected gene flow in *Causonis* and its contribution to gene tree conflicts. **a** A cladogram based on the 23taxa-810nu dataset with nodes colored by reticulation index, showing the effects of gene flow. **b** A cladogram with nodes colored by population mutation parameter theta, showing the effects of ILS. **c** A cladogram with nodes colored by BS values, showing the nodal recovery in simulated gene trees. **d** A cladogram with nodes colored by gene concordance factors, showing the level of gene tree variations. Percentages of gene tree variation ascribed to gene flow, ILS, and gene tree estimation error are indicated beside the arrows. **e** Introgressions in *Causonis* estimated with SNaQ based on the 8taxa-810nu dataset. Hybrid edges with BS values are denoted by blue dashed lines. Minor inheritance probabilities and BS values are shown in blue and black numbers along the edges, respectively. The edge lengths are shown in coalescent units, with the length of each terminal branch set as 1

The consistent patterns of cyto-nuclear discordance and gene tree conflicts showed that it is inadequate to explain gene tree conflicts by either ILS or insufficient phylogenetic signal alone. The relative contributions of gene flow, ILS, and gene tree estimation error in causing gene tree conflicts were then further quantified using the Lindeman, Merenda, and Gold (LMG) method. The analysis showed that gene flow, ILS, and gene tree estimation error can collectively explain 75.6% of the total gene variations across the internal branches (Fig. 3a–d and Additional file 2: Fig. S4). Among these factors, gene flow was the dominant factor, accounting for 45.7% of observed gene tree variations. Notably, most nodes within the core *Causonis* clade showed an above-average level of reticulation index, indicating a greater contribution of gene flow in this clade than in others (Fig. 3a). Surprisingly, ILS, a ubiquitous cause of phylogenetic discordance, only accounted for 3.0% of gene tree variations. Two nodes showed a high level of ILS (i.e., the stem node of the core *Causonis* clade and the crown node of the *C. medogensis*-*C. montana* lineage; Fig. 3b), which also exhibited short internal branches (Fig. 1). Gene tree estimation error accounted for 26.9% of gene tree variations (Fig. 3c). Interestingly, there was a strong correlation between the contributions of gene tree estimation error and gene flow (Fig. 3a, c), likely because gene flow often leads to short internal branches that increase gene tree estimation error substantially [45].

*D-*statistics representing the deviations of derived polymorphisms shared between lineages showed that all the species combinations with a significant *P*-value < 0.05 and *Z*-score > 3 had an average *D* = 0.30, with a maximum value of 0.59, suggesting frequent hybridizations between lineages represented by *C. daliensis* (CPG41826) and *C. corniculata* (CPG41978) (Additional file 1: Table S8). In general, higher *D*-statistics have been observed within the core *Causonis* clade, suggesting frequent hybridizations in the recent divergence of the genus (Additional file 2: Fig. S5), which is consistent with previous findings [31]. On the contrary, no strong signal of hybridization was detected between species in the early-diverged lineage (Additional file 2: Fig. S5). Although the scarce hybridization in the early-diverged lineage may be caused by a lack of contact, species with overlapping distribution ranges (e.g., *C. australasica* and *C. clematidea*) also showed little signal of hybridization, which may be due to more rigorous biological or geographical reproductive isolation.

The phylogenetic network inference was conducted using Species Networks applying the Quartets (SNaQ) method on nuclear genes of the selected seven species that represent the main clades of *Causonis* and one outgroup species from *Pseudocayratia* (8taxa-810nu dataset, see Additional file 1: Table S7)*.* The slope of negative log pseudolikelihood (− logplik) score suggested two hybridization events (Additional file 2: Fig. S6). One introgression occurred between *C. fugongensis* and the ancestor of the japonica-corniculata clade plus *C. fugongensis*, with a BS value of 84%, where the ancestor contributed 23.6% of genetic material to *C. fugongensis* (Fig. 3e). It is worth noting that such gene flow between ancestors and their descendants is unlikely to occur, and the phylogenetic relationships among *C. fugongensis*, *C. japonica* var. *pseudotrifolia*, and *C. daliensis* also differed from the species tree. One possible explanation is that there was a now-extinct “ghost lineage” diverging from the stem of the clade consisting of the three species mentioned above [46], and contributing genetic materials to *C. fugongensis*. But considering the abundant hybridization signals in this clade, it is more likely that this gene flow is an artifact caused by the limitation of SNaQ on detecting multiple introgression events in a small group of species [47]. The phylogenetic network also showed gene flow from *C. ciliifera* to the ancestor of the core *Causonis* clade (5.34%), with a BS value of 70% (Fig. 3e). This gene flow may account for the nuclear gene tree conflicts in the early-diverged lineage of *Causonis* and can provide a clue for the difficulties in identifying the phylogenetic position of *C. ciliifera* using the 136taxa-79pd dataset.

### Whole genome duplication events in the evolutionary history of *Causonis*

**Fig. 4:**
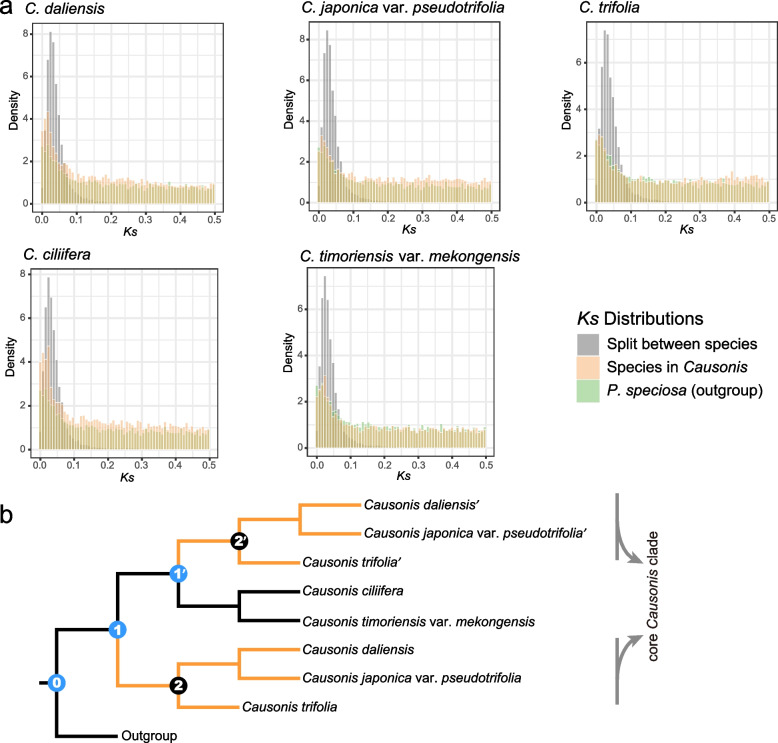
Inferred whole genome duplication events in *Causonis* based on transcriptome data. **a** Distribution density of *Ks* distances of ortholog pairs between species of *Causonis* and the outgroup *Pseudocayratia speciosa*, and the distribution density of *Ks* distances of paralog pairs within the corresponding species, indicating genome duplications after the split between *Causonis* and *Pseudocayratia*. **b** Optimal MUL-tree inferred from GRAMPA analyses, using homologous gene trees against multispecies coalescent tree. Orange branches denote the allopolyploid origin of the core *Causonis* clade. Nodes marked in blue show the positions where polyploidization might have occurred, as suggested by gene tree reconciliation on the single-labeled tree

The WGD events in *Causonis* were explored by analyzing synonymous distances (*Ks*) distributions and mapping gene trees to the inferred species tree. All the species showed four optimal mixing components with the *Ks* mean around 0.03, 0.2, 0.6, and 1.5, respectively (Additional file 1: Table S9 and S10; Additional file 2: Fig. S7). The fourth component corresponds to the whole genome triplication in the core eudicots [48], and the other three components suggested three potential WGD events in *Causonis* and *Pseudocayratia*. *Ks* plots of interspecific orthologs were also generated to determine the relative timing of WGD events and species divergence. The *Ks* peaks representing the divergence of *Pseudocayratia* and *Causonis* species were always older than *Ks* peaks of intraspecific paralogs (Fig. 4a), suggesting that two WGD events (with a *Ks* peak at around 0.03) might have independently occurred in *Causonis* and *Pseudocayratia*. For other species pairs within *Causonis*, no significant sequential differences have been detected between intraspecific and interspecific *Ks* peaks (Additional file 2: Fig. S8), which may be due to the very young age of WGD events and the susceptibility of *Ks* peaks to substitution rate heterogeneity across genes.

The homolog trees were mapped to the coalescent-based species tree to identify gene duplication clusters using the least common ancestor (LCA) reconciliation method. Of all the nodes on the species tree, Node 0 and Node 1 in Fig. 4b and Additional file 2: Fig. S9 met the criteria for being considered to have experienced a WGD event (see “Methods”). Node 0 had 4134 duplicated genes (gene duplication ratio = 22.74%), of which 1781 duplicated genes were of the (AB)(AB) type (Additional file 1: Table S11; Additional file 2: Fig. S9), representing WGD events shared by *Pseudocayratia* and *Causonis*. Node 1 had 730 duplicated genes (gene duplication ratio = 5.54%), of which 573 duplicated genes were of the (AB)(AB) type (Additional file 1: Table S11; Additional file 2: Fig. S9), representing a WGD event shared by all the *Causonis* species. Interestingly, Node 2 in Additional file 2: Fig. S9 (Node 2 and Node 2’ in Fig. 4b) showed an extremely low number of gene duplications and gene duplication ratio, with only 19 duplicated genes and a gene replication rate of 0.38% (Additional file 2: Fig. S9).

Because of the strong signal of hybridization detected in *Causonis*, the possibility of allopolyploidy needs to be considered. Allopolyploidy occurs when an individual receives sets of chromosomes from its parents of different species through hybridization, and therefore, the duplicated genes of the species are more similar to orthologs, as they are related by a speciation event tracing back to their most recent common ancestor [49]. Gene trees were mapped to multi-labeled trees (MUL-trees) with the LCA reconciliation method to identify the most parsimonious tree, and distinguish allopolyploidy from autopolyploidy. The MUL-trees revealed an allopolyploid origin of the core *Causonis* clade, resulting from hybridization between the ancestors of the *C. timoriensis* var. *mekongensis*-*C. ciliifera* lineage and an extinct or unsampled lineage (Fig. 4b and Additional file 1: Table S12).

The polyploidy events shown by LCA reconciliation to MUL-trees were consistent with the results of LCA reconciliation to standard trees and *Ks* distribution plots, although they identified genome duplication events at different positions of the species tree (Fig. 4 and Additional file 2: Fig. S9). In a scenario of allopolyploidy, LCA reconciliation will map gene duplication events to the most recent common ancestor of the two parental lineages, which may be assumed as the nodes where WGD events have occurred. This is exactly what the LCA reconciliation analysis from this study unveils; the polyploidy event was identified as occurring in the ancestor of the whole genus instead of the core *Causonis* clade (Fig. 4b). This may also result in particularly low gene duplication and gene duplication ratio at the ancestor of the core *Causonis* (Node 2 and Node 2’ in Fig. 4b, Node 2 in Additional file 2: Fig. S9), where the duplicated genes might have been mapped to an older node due to the mixing components of the two parental lineages. Hybridization also makes the *Ks* peaks of genes duplicated through allopolyploidy seem older, leading to more difficulty in comparing the relative timing of genome duplication and speciation events. In the *Ks* plot for ortholog pairs of *Causonis* species, it can be seen that the peaks representing speciation and those showing genome duplication events almost overlapped (Additional file 2: Fig. S8).

### Functional enrichment of duplicated genes

The transcriptomes were annotated and gene ontology (GO) term enrichment analyses were performed on the identified duplicated genes for the three species in the core *Causonis* clade: *C. daliensis*, *C. japonica* var. *pseudotrifolia*, and *C. trifolia*, in order to explore whether genes duplicated through WGD events have preserved some functions that may benefit survival. All three species showed an enrichment of genes related to immune and reaction to biotic stimulation (especially to pathogens), such as programmed cell death induced by symbiont and plant-type hypersensitive response in all three species, and regulation of immune response in *C. daliensis* and *C. trifolia* (Additional file 2: Fig. S10). The over-represented genes indicated an increase of disease resistance through programmed cell death in the species of the core *Causonis* clade obtained through allopolyploidy, which may facilitate the expansion of their distribution range. In addition, genes were also significantly enriched in functional categories such as ADP binding, response to red light and regulation of transmembrane transport, which may also help the species survive in a hypertonic environment during dispersal (Additional file 2: Fig. S10).

### Biogeographic history of *Causonis*

**Fig. 5:**
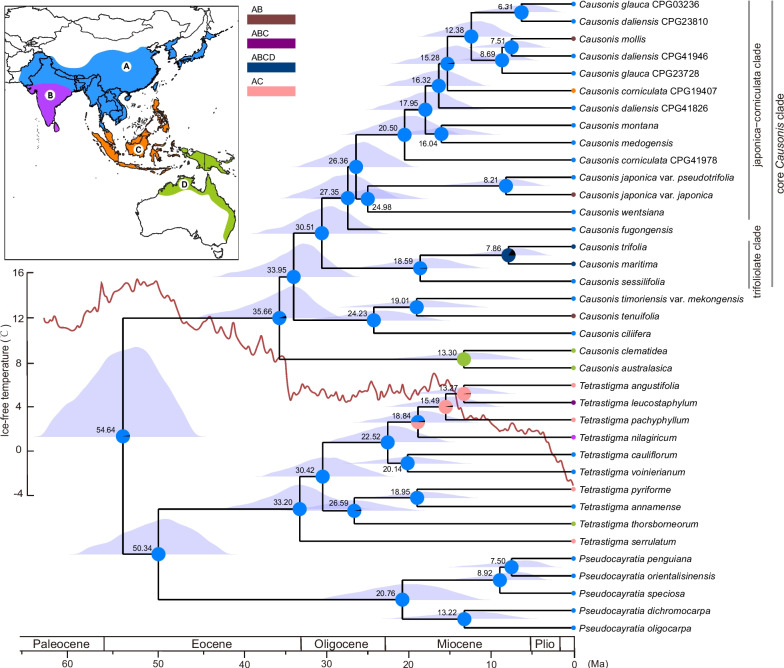
Ancestral area reconstructions for *Causonis* by BioGeoBEARS using the chronogram based on the 76taxa-50nu dataset. Mean node ages are displayed at the corresponding nodes, and posterior distributions were plotted to represent the 95% highest posterior density confidence interval of divergence times. The pie charts indicate the relative possibilities of ancestral areas estimated. A, continental Asia (including the East Asian monsoon region, Sino-Himalaya, and Indochina); B, the Indian subcontinent; C, the Malesian region (excluding New Guinea); and D, Australasia (including continental Australia and New Guinea). The red line represents the global temperature changes (adapted from Westerhold et al. [50]). For concision, outgroup except for *Tetrastigma* and *Pseudocayratia* are not shown. Ma, million years ago

The divergence time was estimated with clock-like nuclear genes (76taxa-50nu dataset) and plastid genes (76taxa-79pd dataset) (Additional file 1: Table S7), and the resulting time trees were used to conduct ancestral area reconstruction with the best biogeography models based on the AICc value (Additional File 1: Table S13). According to the 76taxa-50nu dataset, the crown group of *Causonis* originated from continental Asia in the late Eocene (35.66 Ma; Fig. 5 and Additional file 2: Fig. S11). The clade including *C. timoriensis* var. *mekongensis*, *C. tenuifolia*, and *C. ciliifera* diverged from the core *Causonis* clade in the early Oligocene (33.95 Ma), and the crown group of the core *Causonis* clade formed around 30.51 Ma (Fig. 5 and Additional file 2: Fig. S11). The ancestor of *C. clematidea* and *C. australasica* might have also dispersed to Australasia during this period. The crown group of the japonica-corniculata clade may have originated in the Oligocene (26.36 Ma), and most *Causonis* species in this clade diverged during the Miocene, especially around the Miocene Climatic Optimum (Fig. 5 and Additional file 2: Fig. S11). The range expansion of species occurred mostly after the Miocene, with four independent dispersals to India and one to Indonesia (Fig. 5). In general, the 76taxa-79pd dataset suggested similar origin time and dispersal patterns of *Causonis* as the 76taxa-50nu dataset, but it showed more recent divergences of most lineages, especially in the japonica-corniculata clade, where many taxa diverged after the late Miocene (Additional file 2: Figs. S12 and S13).

### Species distribution modeling of widely vs. narrowly distributed species

**Fig. 6:**
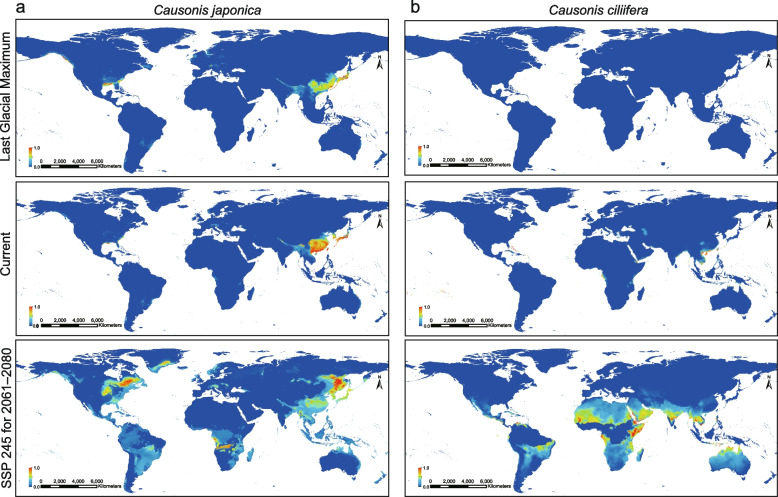
The global potential distribution of **a** *Causonis japonica* and **b** *C. ciliifera* under the LGM (0.021 Ma), the current condition (1970–2000), and the future (2061–2080) on the shared socioeconomic pathway (SSP) 245 scenario, modeled using MaxEnt. The color panel in the bottom left refers to the level of climatic suitability, which increases with color from blue to red

Current potential distribution and scenarios of the past (the Last Glacial Maximum, LGM) and future (the 2061–2080) of representative widespread and narrowly distributed species were predicted by species distribution modeling, using carefully checked and filtered distribution records (Additional file 1: Table S14). The AUC values for climatic modeling were above 0.97, indicating good performance of the predictive models. For *C. japonica*, a well-known widespread species in *Causonis*, the predicted climatic suitability areas under the current condition were consistent with its empirical distribution areas, mainly in the subtropical monsoon climatic zone of East Asia, with small climatic suitability areas in eastern North America (Fig. 6a). During the LGM, the climatic suitability area of *C. japonica* decreased and shifted southward to the exposed continental shelf in the contemporary Pacific Ocean (Fig. 6a). In 2061–2080, the suitable habitats of *C. japonica* were predicted to extend northward in both East Asia and northeastern North America (Fig. 6a), indicating a more severe potential invasion of the species in North America in the near future.

For the narrowly distributed species, *C. ciliifera*, the predicted climatic suitability areas under the current condition were restricted to the coastal areas of southern East Asia and the islands near the continent (Fig. 6b), indicating a preference for heat and humidity. Almost no climatic suitability areas were identified during the LGM (Fig. 6b). In 2061–2080, with increasing global warming, the suitable habitats of *C. ciliifera* were estimated to extend to Indochina, India, and Australia, but its suitability in the current distribution area was found to decline. Geographic barriers, such as the Truong Son Ridge and the Luzon Strait, have been observed between the future suitable areas and the current distribution areas (Fig. 6b).

## Discussion

This study presents a robust reticulate evolutionary history of *Causonis*, which resolves its phylogenetic relationships and identifies hybridizations and WGD events based on a comprehensive analysis using both plastid and nuclear datasets. We confirmed well-supported cyto-nuclear discordances and nuclear gene tree conflicts within *Causonis* (Figs. 1 and 2), as reported previously [30, 31]. Hybridizations are identified from deep to shallow branches in *Causonis*, with a highest frequency in the japonica-corniculata clade (Fig. 3 and Additional file 2: Fig. S5). Gene flow is found to be the dominant factor that accounts for 45.7% of observed gene tree variations, compared to ILS (3.0%) and gene tree estimation error (26.9%) (Fig. 3a–d). We infer an allopolyploid origin of the core *Causonis* clade, which is most likely derived from the hybridization between extinct early-diverged lineages of *Causonis*. We also find increased stress-related gene copies in representative species of the core *Causonis* clade probably resulting from allopolyploidization. The well-explored reticulate evolutionary history of *Causonis* under a robust biogeographic framework reveals distinct hybridization and WGD modes in lineages with wide vs. narrow distribution ranges.

### Allopolyploid origin of the core *Causonis*

Based on the reticulate evolutionary history of *Causonis* in a robust spatial–temporal framework, we illustrate a possibility of how the excessive disparity in species distribution ranges between the early-divergent and core *Causonis* clades have formed. After the divergence of *Causonis* from the *Tetrastigma*-*Pseudocayratia* clade in the early Eocene, the early-divergent lineages of *Causonis* are inferred to migrate from continental Asia to Australasia (Fig. 5 and Additional file 2: Fig. S13), following the trend of the Great Asiatic Floristic Interchanges 2 [51]. The hybridization event leading to the allopolyploid origin of the core *Causonis* clade might have occurred during the floristic exchanges (Fig. 4)*.* The Oi-1 Glaciation in the early Oligocene likely caused extensive extinction in most lineages of *Causonis*, as suggested by a 10–20-million-year time lag between the crown and stem groups of *Causonis* (Fig. 5 and Additional file 2: Figs. S11–13). The ancestor of the core *Causonis*, equipped with duplicated genomes, might have survived the glaciation and flourished in continental Asia during the Miocene, resulting in a clade with higher species richness and more widespread species (Fig. 5). The increase in genetic diversity and higher gene dosage resulting from allopolyploidization might have promoted their adaptation to changing environments, as suggested by the enriched stress-related GO terms (Additional file 2: Fig. S10). Previous studies reported more robust morphology and easier asexual reproduction in polyploid species [52], which may have further promoted their adaptation and range expansion. The extremely wide distribution range of some species from the core *Causonis* clade, such as *C. japonica* and *C. trifolia*, might have been enhanced by the combined effect of allopolyploid origin and recent polyploidization.

The japonica-corniculata clade shows frequent recent hybridizations, as suggested by the reticulate index, *D*-statistics, and phylogenetic network (Fig. 3 and Additional file 2: Fig. S5). The plastid data suggest much later divergence times in the japonica-corniculata clade, with many taxa diverging after the late Miocene, which are much younger than those inferred by the nuclear dataset (Fig. 5 and Additional file 2: Figs. S11–13). While this can be explained by the non-constant substitution rate in plastid genes due to smaller effective population size inferred from plastids [53, 54], it can also indicate plastid capture, revealing further evidence for introgression, as plastid genomes generally preclude recombination and retain similarity between hybridized lineages [55]. The fluctuating temperature declines since 5 Ma (Fig. 5) might have facilitated hybridization by promoting migration and increasing the possibility of secondary contacts. While frequent gene flow between species could be an outcome of range expansion [56], hybridization can consolidate the wide distribution range of species by maintaining or increasing the size of edge populations [11, 57]. Another mechanism by which hybridization promotes species range expansion involves the introgression of adaptive alleles from resident species. However, obtaining direct evidence of novel traits acquired through a specific gene flow is difficult, due to the overlapping ecological niches and ubiquitous gene flow among species in the japonica-corniculata clade [30] (Fig. 3 and Additional file 2: Fig. S5). Future studies with denser population-level sampling may better quantify relationships among hybridization, morphological innovation, and distribution range expansion.

### Species distribution modeling and conservation implications

Species distribution modeling in this study reveals that the widespread *C. japonica* and the narrowly distributed *C. ciliifera* may have experienced different biogeographic past and may be expected to face different conservation issues. For the widespread *C. japonica*, recognized as invasive in some regions [33, 34], the predicted suitable habitats during LGM seem to be only moderately reduced compared with its current distribution (Fig. 6 and Additional file 2: Fig. S15), indicating persistence within Quaternary fluctuating environments to a large extent. The significant northward expansion of suitable habitats in 2061–2080, especially in North America, indicates a much wider distribution of *C. japonica*, which may become a conservation concern (Fig. 6a). It is also worth noting that the future suitable habitats of *C. japonica* also expand southward, even possibly to Indonesia and Australia (Fig. 6a). The potential rapid invasion of *C. japonica* may put the indigenous *Causonis* species at risk of extinction due to demographic or genetic swamping [58]. Therefore, besides restraining further dispersal of *C. japonica* and drafting rapid-response plans for population control, detection of the actual level of hybridization in the new colonies of *C. japonica* should also be considered [59].

For *C. ciliifera*, a species endemic to the coastal region in Hainan, China, and Vietnam, the dramatic shifts in the climate suitability areas suggest its vulnerability to environmental change (Fig. 6b). During the LGM, the suitable habitats for *C. ciliifera* seem to have been dramatically decreased (Fig. 6b), suggesting that small populations of *C. ciliifera* might have survived only in refugia. While the prediction of suitable habitats in 2061–2080 seems to indicate the possibility of *C. ciliifera* becoming widespread, it should be noticed that the suitability in its current specific distribution areas is declining (Fig. 6b). Therefore, its future largely depends on the ability to transcend geographic barriers between predicted fitness areas and current distribution areas (Fig. 6b). If *C. ciliifera* fails to disperse, it may become endangered due to limited adaptation ability to varying environments. Conservation actions should be taken to save populations and habitats of narrowly distributed species, such as *C. ciliifera*. Monitoring of population dynamics and artificial transplanting, to some extent, may reduce their extinction due to the bottleneck effect of small populations.

It should be noted that the suitable habitats predicted based merely on climatic factors may not necessarily represent the actual distribution of a species, which usually results from complex interplay between environmental and biotic factors. However, the predictions indeed reflect different trends in the distribution shift of wide- and narrow-ranged species. Our results support conclusions from previous research that widespread species tend to expand their range, while narrowly distributed species tend to show the opposite trend [60–62]. Narrowly distributed species typically have specialized adaptation to unique habitats, whereas widespread species are more adaptable and competitive [63, 64]. Global climate change not only alters the habitats of narrowly distributed species, making them less adapted to local environment, but also exposes them to biotic pressures of competition with widespread species [65]. This makes the narrowly distributed species easily replaceable by widespread species, leading to biotic homogenization and the loss of unique functions supported by narrowly distributed species in local ecosystems [61, 66–68]. Therefore, protecting narrowly distributed species and their habitats, as emphasized in this study, represents an effective approach to slowing biodiversity loss in the face of changing global climate. For species with complex evolutionary histories, we propose that conservation strategies should consider both their genetic and biogeographic properties.

## Conclusions

This study has revealed a reticulate evolutionary history of *Causonis*, involving hybridization and WGD events based on a dense sampling of species and a large amount of plastid and nuclear data. In previous studies, inferring hybridization events has been challenging, often due to multiple factors such as gene flow, ILS, and gene tree estimation error that combined to generate conflicting phylogenetic signals. The problem can be more perplexing when frequent gene flow occurs within a small clade [69–72]. The detection of WGD events is also a concerning problem, for which popular methods such as the *Ks*-based determination and LCA reconciliation mapping gene trees to a standard species tree have been developed [73–75]. However, both methods show shortcomings in detecting allopolyploidization events [76, 77], as the study presented. By evaluating gene alignment informativeness and integrating results from the reticulate index, *D*-statistic test, and network inference, the effects of hybridization events were distinguished from other factors and revealed hybridizations in both the deep and shallow branches of *Causonis.* In addition, this study identified an allopolyploidization event in *Causonis* mainly based on the evidence from LCA reconciliation of gene trees to multi-labeled species trees, besides the two popularly used methods for WGD detection. The core *Causonis* clade was inferred to have an allopolyploid origin, which might have facilitated the accumulation of stress-related gene copies. Combining the results of divergence time estimation and ancestral area reconstruction with the reticulate evolutionary history, this study inferred that hybridization and WGD events might have promoted species to survive in severe environments (e.g., glaciation). Species distribution expansion in the core *Causonis* clade might be achieved through evolutionary innovation and edge population maintenance, and thus led to species distribution range disparity in the genus. This study shows a case of different hybridization and whole genome duplication modes in lineages with wide vs. narrow species’ geographic ranges, which helps understand the evolution of species’ distribution range. We also emphasize the importance of incorporating species’ genetic properties and specific biogeographic histories into conservation practice to decrease the loss of narrowly distributed species in competition with widespread species.

## Methods

### Taxon sampling and sequencing

We sampled 82 individuals of *Causonis*, representing 16 species and 3 varieties of the genus. To use reliable calibration points in Vitaceae for divergence time estimation, 52 species from other genera of Vitaceae were also sampled as outgroups, which covered all the main clades of the family. Two species of Leeaceae, *Leea indica* and *L. rubra*, were used as outgroup to construct the phylogeny of Vitaceae.

Of the 136 sampled individuals, fresh leaves of six individuals, including five individuals of *Causonis* (*C. ciliifera*, CPG41949; *C. daliensis*, CPG41826; *C. japonica* var. *pseudotrifolia*, LC-10; *C. timoriensis* var. *mekongensis*, DBH20210706; *C. trifolia*, CPG71908) and one individual of a closely related species, *Pseudocayratia speciosa* (LC-07), were used for total RNA extraction, library construction, and sequencing. Paired-end reads of 150 bp for all samples were generated by an Illumina HiSeq2500 sequencer. For the other 130 individuals, whole genome sequencing (WGS) was conducted for 108 individuals. Library construction and sequencing were based on DNA extracted from silica gel-dried leaves. Paired-end cDNA or genomic DNA libraries were generated, and for each individual, 150-bp reads were generated by an Illumina HiSeq2500 sequencer for a total of 6–10 G. WGS data of the other 22 species were downloaded from NCBI. Voucher and data information of all individuals sampled in this study are shown in Additional file 1: Tables S2 and S3.

### Transcriptome assembly and ortholog inference

For raw reads obtained from transcriptome sequencing, low-quality reads and low-quality bases at read ends were trimmed, and adapter artifacts were filtered out from the raw reads using Trimmomatic v.0.39 [78] with the following parameters: SLIDINGWINDOW:4:20 LEADING:20 TRAILING:20 MINLEN:50. Potential organelle reads were filtered using Bowtie2 v.2.4.5 [79]. Transcripts were de novo assembled using Trinity v.2.5.1 [80, 81] with default settings. For the six transcriptomes, assembly quality analyses using TransRate v.1.0.1 [82] and filtered out badly supported transcripts with the best contig score cut-off that maximizes the assembly score. The completeness of transcriptome assembly was assessed using BUSCO v.5.2.2 [83]. The coding sequences of the longest isoform of each gene were predicted and translated by TransDecoder v.5.5.0 [80], and the redundancy was removed by CD-HIT v.4.7 [84] with a threshold value of 0.98. Based on the output of CD-HIT using OrthoFinder v.2.5.4, 2143 “single-copy orthologous genes” from the six transcriptomes were identified [85] with default settings. We then used reciprocal BLASTN v.2.5.0 [86] to remove genes with multiple hits within the same species or with an E-value > 10^−6^, retaining 1377 genes. To avoid misleading phylogenetic signals, we removed orthologs showing potential long-branch attraction and saturation using TreSpEx v.1.1 [87], retaining 827 genes. These genes were aligned with MAFFT v.7.310 [88] using the L-INS-i algorithm and trimmed by trimAl v.1.4 [89] with the option “automated1”, and alignments < 450 bp were removed. With the remaining genes as targets, we extracted coding sequences of other 130 sampled individuals from WGS data using HybPiper pipeline v.1.3.1 [90] with the “paralog_investigator” option. All the extracted sequences, combined with genes generated from transcriptome data, were aligned using MAFFT, and homolog trees were constructed using RAxML v.8.2.12 [91] with a GTRCAT model and 100 bootstrap replicates. Finally, orthologs were inferred using the “monophyletic outgroup” strategy [92] that filtered single-copy orthologs by only keeping single-copy and monophyletic taxa groups. A total of 810 orthologs were retained and designated as the 136taxa-810nu dataset (Additional file 1: Table S7). All the alignments of 810 orthologs were manually checked.

### Plastome assembly and annotation

Plastomes of 125 individuals with their WGS data sequenced were assembled using GetOrganelle v.1.7.1 [93] with the plastid genome of *Vitis vinifera* (NC007957) as the reference. The assembled plastomes were then annotated using PGA [94] with the plastomes of *Ampelopsis humulifolia* (NC042236), *Tetrastigma hemsleyanum* (NC029339), and *Vitis vinifera* as references, and the annotations were manually checked in Geneious Prime v.2021.2.2 [95]. A total of 79 protein-coding sequences (CDS) regions were used as targets for HybPiper pipeline to extract the correspondents from the WGS data. The concatenated alignment including 79 CDS for all 136 sampled individuals was designated as the 136taxa-79pd dataset (Additional file 1: Table S7). Each CDS region was aligned and trimmed using the same method applied to the nuclear orthologs and the alignments were manually checked.

### Phylogeny reconstruction

Based on the 136taxa-810nu dataset, we used IQ-TREE v.2.1.2 [96] to construct the phylogeny with the maximum likelihood (ML) approach using the concatenated ortholog alignments with 5000 ultrafast [97] under the edge-unlinked partitioned model [98], and the best model scheme was selected by ModelFinder [99]. We also constructed the phylogeny with Bayesian inference (BI) approach using MrBayes v.3.2.7a [100], with the best codon partition scheme selected by PartitionFinder v.2.1.1 [101]. For the coalescent analysis, the multispecies coalescent (MSC) trees were generated using ASTRAL v.5.7.1 [102], with single gene trees constructed by RAxML with GTRCAT model and 100 bootstrap replicates as the input. To avoid the influence of poorly supported clades on MSC tree-based methods, nodes of gene trees were collapsed with a BS value < 10, 40, or 70% to determine the best cut-off for MSC tree inference. For the 136taxa-79pd dataset, we also constructed phylogeny using both the concatenation and coalescent-based methods with the same software and settings as mentioned above.

### Phylogenetic discordance analyses

We first assessed the discordance of single gene trees. To focus on the phylogenetic conflicts among species of *Causonis*, the alignments of the 136taxa-810nu dataset were pruned to retain sequences of individuals representing each species of *Causonis*, and one representative from each subclade was selected for non-monophyletic species. The individual of *P. speciosa* was used as outgroup. The simplified dataset was designated as the 23taxa-810nu dataset (Additional file 1: Table S7). Based on this dataset, gene trees were constructed using RAxML with the GTRCAT model and 100 bootstrap replicates and were used as input for ASTRAL to estimate the MSC species tree. We used SplitsTree v.4.13.1 [103] to visualize gene tree conflicts within *Causonis*, using gene trees with their nodes below the best BS cut-off collapsed to avoid overestimating gene tree conflicts. To quantify the degree of gene tree discordance on each node, the number of conflicting or concordant bipartitions was calculated, and the internode certainty all (ICA) values using the Phyparts pipeline [104], where gene trees with a BS support value > 70% on the corresponding node, were mapped against the ASTRAL species tree. We also calculated gene concordance factors (gCF) using IQ-TREE, with the ASTRAL species tree as the mapping tree. To distinguish strong conflicts from weakly supported branches, we performed Quartet Sampling (QS) analysis [105] on the ASTRAL species tree with 1000 replicates, which generated scores to assess the confidence (QC, Quartet Concordance), consistency (QD, Quartet Differential), and informativeness (QI, Quartet Informativeness) of internal branches.

The relative contributions of three possible factors responsible for gene tree conflicts, i.e., hybridization, gene tree estimation error, and incomplete lineage sorting (ILS) were assessed, as they have been regarded as primary factors of phylogenetic discordance [69, 71]. The assessment was conducted with a regression model, following the methods of Cai et al. [71]. The dependent variable, i.e., gene tree variation, was denoted by gCF calculated with IQ-TREE. Introgression can be detected by the deviation from the MSC model based on the frequency of triplets, which are the possible topologies of three species: ((A,B),C), ((A,C),B) and ((B,C),A). Under the impact of ILS, the two minor discordant triplets will occur at the same frequency, but introgression can cause the difference in the frequency of two minor triplets. The reticulate index was used to quantify the intensity of gene flow at each node, which was defined as the percentage of triplets showing introgression among all the triplets associated with the node. We first ran ASTRAL on the bootstrap ML gene trees to obtain bootstrap species trees, and then simulated gene trees with each bootstrap species tree under the MSC model using R package Phybase v.1.4 [106]. For each node, triplet frequency in the empirical gene trees and simulated gene trees was counted, then the nodes with significantly unbalanced triplets were identified and reticulate index was calculated with the scripts used in Cai et al. [71].

To further estimate gene flow between species, the *D-statistics* were calculated for all the possible combinations of three species in the 23taxa-810nu dataset, which reflects site patterns (ABBA or BABA) for a specific quartet that includes three ingroup species and one outgroup species, i.e., (((A,B),C),O). When *D-statistics* significantly deviated from 0, it indicates introgressions between B and C (ABBA pattern, *D* > 0) or A and C (BABA pattern, *D* < 0). To test hybridizations between all the *Causonis* species, *D-statistics* were calculated using the “Dtrios” command in Dsuite v.0.4 r41 [107] based on the concatenated 23taxa-810nu dataset, with the ASTRAL species tree used to specify the phylogenetic relationships of individuals. To estimate the level of ILS, we calculated the population mutation parameter “theta” of each internal branch by dividing the branch length into mutation units (obtained from the ML tree) by the length in coalescent units (obtained from the MSC species tree). To quantify the effects of gene estimation error, we simulated 810 gene alignments under the GTR model with the ASTRAL species tree using Seq-Gen v.1.3.4 [108]. The alignments were simulated with a sequence length of 1488, the mean length of empirical gene alignments. Then we calculated how often each node of the empirical species tree was recovered by gene trees constructed from the simulated alignments using RAxML with the “-f b” option. Finally, we used the linear regression model method implemented in the R package Relaimpo [109] to assess the relative importance of hybridization, ILS, and gene tree estimation error.

### Phylogenetic network inference

To identify gene flow between major clades, we selected seven individuals representing the major clades of *Causonis* and one outgroup species (*P. speciosa*) to conduct phylogenetic network inference. As gene tree conflict assessments suggest a high level of introgressions among *C. mollis*, *C. daliensis*, *C. glauca*, *C. corniculata*, *C. medogensis*, and *C. montana*, we only selected one individual of *C. daliensis* from this clade with perplexing phylogeny to minimize the influence of multiple introgressions on phylogenetic network inference with SNaQ. The 136taxa-810nu dataset was pruned to retain only eight individuals (8taxa-810nu, see Additional file 1: Table S7). Phylogenetic network inference was conducted using SNaQ implemented in Julia package PhyloNetworks [110] on the 8taxa-810nu dataset, with the MSC species tree as a starting tree. The optimal network is inferred by calculating the maximum pseudolikelihood of a network from four-taxon concordance factors (CFs), which were generated following the Tree Incongruence Checking in R (TICR) pipeline [111] with ML gene trees constructed by RAxML with 100 bootstrap replicates under GTRCAT model. To determine the best maximum hybrid node number (hmax) for each possible hmax from 0 to 5, we ran SNaQ for 100 searches, and the best hmax was selected based on the slope of a plot of -logplik against hmax. The network generated under the best hmax was used as a starting network for bootstrap analysis with 100 replicates, and 100 SNaQ searches were conducted for each replicate.

### Whole genome duplication and gene duplication analyses

We detected WGD events by analyzing the distribution of synonymous distances (*Ks*) from the six transcriptomes using wgd v.1.1 [112]. Within each species, we performed all-against-all BLASTP and MCL clustering to build gene family with the command “wgd mcl” [113, 114] to calculate the *Ks* distribution of paralog pairs from the gene family alignments constructed by MAFFT with the command “wgd ksd” [88, 115, 116]. The *Ks* peaks were identified with the R package mixtools v.1.2.0 [117], and the optimal number of mixing components was determined from 2 to 6, using likelihood ratio test statistic in mixtools, with 500 parametric bootstrap replicates. The null hypothesis of *k*-components vs. the alternative hypothesis of (*k* + 1)-components were tested until the *P*-value calculated is above 0.05, and when the test terminates, the last *k* is the optimal number of components. To identify the relative timing of WGD events, we calculated the *Ks* distributions between species using wgd and compared the peaks with those in *Ks* distributions of paralogs within species.

We also identified WGD events by assessing gene duplication events with the phylogenetic methods. Orthogroups were identified using OrthoFinder2 with default settings, among which orthogroups including at least four taxa were aligned using MAFFT and were used to construct gene trees using RAxML with the GTRCAT model and 100 bootstrap replicates. The homologous gene trees were mapped to the ASTRAL species tree with an LCA reconciliation method using Tree2GD v.1.0.40 (custom software available from https://github.com/Dee-chen/Tree2gd [118]) to detect the positions of gene duplication (GD) events. Gene duplication was counted only when the node showing paralogy had a BS value above 50%. Nodes meeting the conditions below were considered to have undergone WGD, following Zhang et al. [119]: (1) exhibiting > 500 GDs, of which > 250 GDs are of (AB)(AB) type; (2) exhibiting > 1500 GDs, of which > 100 GDs are of (AB)(AB) type, and the sum of (AB)(AB) type and (AB)A type or (AB)B type exceeds 1000. As *Ks*-based and LCA reconciliation methods may be incorrect for allopolyploids [77], we tested the polyploidy mode with the same set of homologous gene trees using GRAMPA v.1.3.1 [77], which selected the most reliable hypothetic scenario of autopolyploidy or allopolyploidy by reconciling gene trees to different MUL-trees or single-labeled trees. When gene trees are mapped to the MUL-tree, nodes in the gene trees can be identified as duplication nodes or speciation nodes, and gene losses can also be recognized. For each MUL-tree, the reconciliation score is the total number of duplications and gene loss events, and a lower reconciliation score denotes a more reliable topology according to the principle of parsimony.

To determine whether some of the function categories were enriched in duplicated genes, we conducted a gene ontology (GO) enrichment analysis for each species. The best matching ortholog from *Arabidopsis thaliana* for each gene was found by BLAST, and GO terms were assigned to duplicated genes by combining the annotation results of eggNOG-mapper v.2.1.9 [120] and InterProScan 5 [121]. At the node showing inferred WGD events within *Causonis*, gene ontology terms enriched on the duplicated genes identified by Tree2GD were identified using the R package clusterProfiler [122].

### Divergence time estimation

To avoid excessive sampling resulting in overestimation of divergence times, we selected one representative for each species. For non-monophyletic species, one representative was selected for each clade of this species. As the missing rates of gene sequences extracted from the WGS data and transcriptome data vary, we used the 229 single-copy nuclear genes identified from transcriptomes covering the phylogeny of Vitaceae in Wen et al. [123] as targets for HybPiper pipeline to extract nuclear gene sequences of 76 species representatives. Considering the great computation demand in divergence time estimation, to reduce the scale of the dataset, we used Python package SortaDate [124] to retain clock-like genes having discernible information content and the least topological conflicts with the MSC species tree. The selected 50 “best” genes (76taxa-50nu dataset, see Additional file 1: Table S7) were used for divergence time estimation using MCMCTree implemented in PAML v.4.9j package [110], with *Leea indica* and *L. rubra* as outgroup.

The stem age of Vitaceae was constrained as 89.7–113.6 Ma using a secondary calibration point from Magallón and Castillo [125]. We also used two fossil calibrations to constrain the age boundaries of two internal nodes. The confirmed seed fossil of *Ampelocissus parvisemina* from the late Paleocene in North Dakota of North America [126] was used to constrain the crown age of the *Ampelocissus*–*Vitis* clade as 56.8–62.0 Ma. The late Eocene *Vitis glabra* Chandler from the lower Bagshot beds of the London Clay of southern England [127, 128] was used to constrain the minimum crown age of subg. *Vitis* as 35.0 Ma. We followed a conservative approach for all calibration points, where uniform prior distributions with soft bounds were employed, with 2.5% prior density extending above and below the bounds.

MCMCTree was run with the following settings: birth–death model, correlated rates, HKY85 substitution model with an alpha = 0.5, and a pruned ASTRAL MSC species tree was used to constrain the tree topology. The maximum likelihood estimates of branch lengths were estimated, followed by the gradient and Hessian matrices estimated with the usedata = 3 option. Subsequently, the gradient and Hessian matrices with the usedata = 2 option were used to estimate divergence times in MCMCTree. The results of two independent MCMC chains were combined with 20% iterations discarded as burnin, where samples were drawn every 1000 generations and 10,000 samples were collected in total. The effective sample sizes for all the parameters were checked in Tracer v.1.7 to ensure they are > 200.

We also estimated the divergence time based on the simplified 76taxa-79pd dataset (Additional file 1: Table S7) including the same species as the 76taxa-50nu dataset. As the phylogenetic position of *C. ciliifera* cannot be fully resolved with the 136taxa-810nu dataset, we constructed an ML tree with the concatenated 76taxa-79pd dataset using IQ-TREE, with 5000 ultrafast bootstrap and the best model scheme selected by ModelFinder. We used this ML tree to fix the topology during divergence time estimation. The analysis was conducted using MCMCTree with the same calibration points and procedures as described above for the nuclear estimation. All the analyses were performed twice with different seeds to confirm consistency of the results.

### Ancestral area reconstruction

Ancestral area reconstructions were conducted on the 22 representative individuals of *Causonis* in the 23taxa-810nu dataset, combined with outgroup consisting of 15 individuals from *Pseudocayratia* and *Tetrastigma* with clear distribution information available. Based on the distribution information of each species in *Causonis*, we divided the distribution range of the genus into four areas to investigate the dispersal pattern of *Causonis*: (A) continental Asia (including the East Asian monsoon region, Sino-Himalaya, and Indochina); (B) the Indian subcontinent; (C) the Malesian region (excluding New Guinea); and (D) Australasia (including continental Australia and New Guinea). The ancestral area of *Causonis* was estimated using the R package BioGeoBEARS [129] implemented in RASP v.4.2 [130], with the dated phylogenies obtained based on the nuclear or plastome datasets as the input trees, respectively. The analyses were conducted with the ML versions of three biogeographic models: DEC (dispersal-extinction-cladogenesis) [131], DIVALIKE (dispersal-vicariance analysis) [132], and BAYAREALIKE (Bayesian inference of historical biogeography for discrete areas) [133]. For each model, the effects were tested with or without parameter* j* [134], which indicates a founder event. The best model was selected according to the corrected Akaike information criterion (AICc).

### Species distribution modeling

Species distribution information of *Causonis* was collected from GBIF (https://www.gbif.org/ [135]) and CVH (https://www.cvh.ac.cn/ [136]). We carefully filtered the records and removed records that: (1) fall in the open ocean; (2) are poorly geo-referenced (e.g., with equal or zero latitude and longitude values); (3) obviously indicate cultivation (e.g., cultivated in a botanical garden, biodiversity institutions, etc.). Images available from GBIF or CVH were checked to confirm species identity. To thoroughly cover the distribution areas of all species in *Causonis*, we also combined the distribution information from Parmar et al. [30], which contains the most comprehensive records of accurately identified specimens of *Causonis* to date. According to the distribution information, *C. japonica* (including two varieties: *C. japonica* var. *japonica* and *C. japonica* var. *pseudotrifolia*) were selected to represent the widespread species, and *C. ciliifera* to represent the narrowly distributed species.

Climatic layers with a 30-s spatial resolution comprising 19 bioclimatic variables representing conditions of current climate were downloaded from the WorldClim Current Conditions dataset, 1970–2000, v.2.1 (https://www.worldclim.org/data/worldclim21.html/ [137]). To eliminate multicollinearity effects in parameter estimates, pairwise Pearson’s correlation coefficients (*r*) of all the 19 bioclimate variables were calculated and excluded the variables with |Pearson’s *R*|≥ 0.80. For each species, key bioclimate variables that can influence the physiological performance and distribution range of species were selected. For *C. japonica*, the selected bioclimatic variables are annual mean temperature (Bio1), mean diurnal range (Bio2), isothermality (Bio3), max temperature of warmest month (Bio5), annual precipitation (Bio12), precipitation seasonality (Bio15), precipitation of driest quarter (Bio17), and precipitation of warmest quarter (Bio18). For *C. ciliifera*, the selected bioclimatic variables are Bio2, Bio3, max temperature of coldest month (Bio6), mean temperature of wettest quarter (Bio8), precipitation of driest month (Bio14), Bio15, and precipitation of coldest quarter (Bio19). Climate dataset of BCC-CSM2-MR model under the shared socioeconomic pathway (SSP) 245 scenario from 2061 to 2080 was downloaded from the WorldClim database v.2.1 (https://www.worldclim.org/data/worldclim21.html/ [137]), with a 30-s spatial resolution. Paleo-Climate dataset representing climate conditions in the Last Glacial Maximum (LGM, 0.021 Ma) was downloaded from the WorldClim database v.1.4 (https://www.worldclim.org/data/v1.4/worldclim14.html/ [138]), with a 2.5-min spatial resolution. ArcGIS v.10.8 (ESRI Inc., Redlands, CA, USA) were used to obtain suitable climatic layers for species distribution modeling.

We estimated the past, current, and future climatic suitable distributions of *C. japonica* and *C. ciliifera* using species distribution modeling with MaxEnt v.3.3.3 k [139]. Our modeling was set as follows: (1) cloglog output format was used to evaluate distribution probability of the two species; (2) ten independent bootstrap replicates for model testing; (3) jackknife analyses of the regularized gain using the training data were conducted to elucidate the importance of each predictor; and other additional parameters were set to default.

The performance of models was evaluated by receiver operating characteristic (ROC) curves. The area under the ROC curve (AUC) ranges from 0 to 1, and an AUC score higher than 0.7 confirms the validity of the results. Since ROC may not be sufficient to fully evaluate the performance of distribution modeling, we also used a binomial test based on a training omission rate, which is the proportion of training occurrence records among pixels of predicted absences. Models with a training omission rate of less than 17% were used.

### Supplementary Information

**Additional file 1: Table S1.** Information on distribution for species of* Causonis *involved in this study. **Table S2.** Detailed information on individuals of *Causonis *sequenced for whole genome sequencing (WGS) data and transcriptome data. **Table S3.** Sources of the whole genome sequencing (WGS) data and transcriptome data for outgroup species in phylogenetic analyses and divergence time estimation. **Table S4. **A summary of transcriptome sequencing. **Table S5.** A summary of transcriptome assembly. **Table S6. **Parsimony-informative sites of each ortholog alignment used for phylogeny reconstruction. **Table S7.** The datasets used in this study and the analyses conducted on the datasets. **Table S8.** *D*-statistic test results on quartets of the combinations of species from *Parthenocissus*, with *P. speciosa* as the outgroup. **Table S9.** *P*-values in likelihood ratio tests for determining the optimal number of mixing components. **Table S10.** The* Ks* peaks inferred as mixtures of normal distributions. **Table S11. **Gene duplications detected with a least common ancestor reconciliation method on standard species tree using Tree2GD, showing gene duplication type. **Table S12. **Reconciliation scores of multi-labeled or single-labeled tree candidates. **Table S13. **Results of model test in BioGeoBEARS, based on the maximum likelihood tree constructed with the 76taxa-79pd dataset and the multi-species tree constructed with the 76taxa-50nu dataset. **Table S14. **Distribution records of *C. japonica *and *C. ciliifera* used in MaxEnt species distribution modeling.

**Additional file 2:** **Fig. S1.** BUSCO assessment results for the transcriptomes of five *Causonis* species and one *Pseudocayratia* species. **Fig. S2.** Phylogenies of *Causonis* generated with the 136taxa-810nu dataset using the maximum likelihood and Bayesian inference methods.** Fig. S3.** Phylogenies of *Causonis* constructed using multispecies coalescent method with 136taxa-79pd dataset and 136taxa-810nu dataset, showing cyto-nuclear discordance. **Fig. S4.** Relative importance of incomplete lineage sorting, gene tree estimation error, and gene flow in generating gene tree variation. **Fig. S5.** Heatmap of *D*‐statistics in the context of phylogenetic relationships for species of *Causonis*, showing introgression events between species. **Fig. S6.** Negative log pseudolikelihood score profiles obtained by SNaQ in PhyloNetworks for eight species. **Fig. S7.** Distribution density of* Ks *distances from 0 to 3 for five species of *Causonis* and one species of *Pseudocayratia*. **Fig. S8.** Distribution density of *Ks* distances of ortholog pairs between species pairs of *Causonis* and the distribution density of *Ks* distances of paralog pairs within the corresponding species. **Fig. S9.** The inferred gene duplication events by mapping homologous gene tree to multispecies coalescent tree using least common ancestor reconciliation. **Fig. S10. **Enriched GO categories of duplicated genes derived from the allopolyploidization in at least two species of the core *Causonis*.** Fig. S11. **Chronogram of *Causonis* inferred from MCMCTree in PAML package based on the 76taxa-50nu dataset. **Fig. S12.** Chronogram of *Causonis* inferred from MCMCTree in PAML package based on the 76taxa-79pd dataset. **Fig. S13. **Ancestral area reconstructions for *Causonis* with BioGeoBEARS using the chronogram based on the 76taxa-79pd dataset.

## Data Availability

All raw data generated and used for this study have been deposited in the NCBI Sequence Read Archive, under BioProject PRJNA1000651 (SRR25601260 to SRR25601265, SRR25607959 to SRR25608035, and SRR25608202 to SRR25608231, as provided in Additional file 1: Table S2 and Table S3) (https://www.ncbi.nlm.nih.gov/bioproject/PRJNA1000651/) [140]. The accession numbers of downloaded data are in Additional file 1: Table S3. Detailed information on sequenced individuals of *Causonis* is in Additional file 1: Table S2, and the voucher specimens used in this study are deposited at the PE herbarium.

## Abbreviations

WGD: Whole genome duplication
ILS: Incomplete lineage sorting
BUSCO: Benchmarking universal single-copy orthologs
WGS: Whole genome sequencing
ML: Maximum likelihood
BI: Bayesian inference
MSC: Multispecies coalescent
BS: Bootstrap support
PP: Posterior probability
ICA value: Internode certainty all value
LMG method: Lindeman, Merenda and Gold method
SNaQ: Species Networks applying the Quartets
−logplik: Negative log pseudolikelihood
*Ks*: Synonymous distances
LCA: Least common ancestor
MUL-trees: Multi-labeled trees
GO: Gene ontology
AICc: Corrected Akaike information criterion
Ma: Million years ago
LGM: Last Glacial Maximum
AUC: Area under the curve
SSP: Shared socioeconomic pathway
CDS: Protein-coding sequences
gCF: Gene concordance factors
QS: Quartet sampling
QC: Quartet concordance
QD: Quartet differential
QI: Quartet informativeness
CF: Concordance factor
TICR pipeline: Tree Incongruence Checking in R pipeline
GD: Gene duplication
ROC: Receiver operating characteristic
