# Supplementary material for: Distinct hybridization modes in wide- and narrow-ranged lineages of Causonis (Vitaceae)
Source: BMC Biol. 2023 Oct 9;21:209. doi: 10.1186/s12915-023-01718-8 (PMC10561429; doi:10.1186/s12915-023-01718-8)

**Figure S1.** BUSCO assessment results for the transcriptomes of five *Causonis* species and one *Pseudocayratia* species.


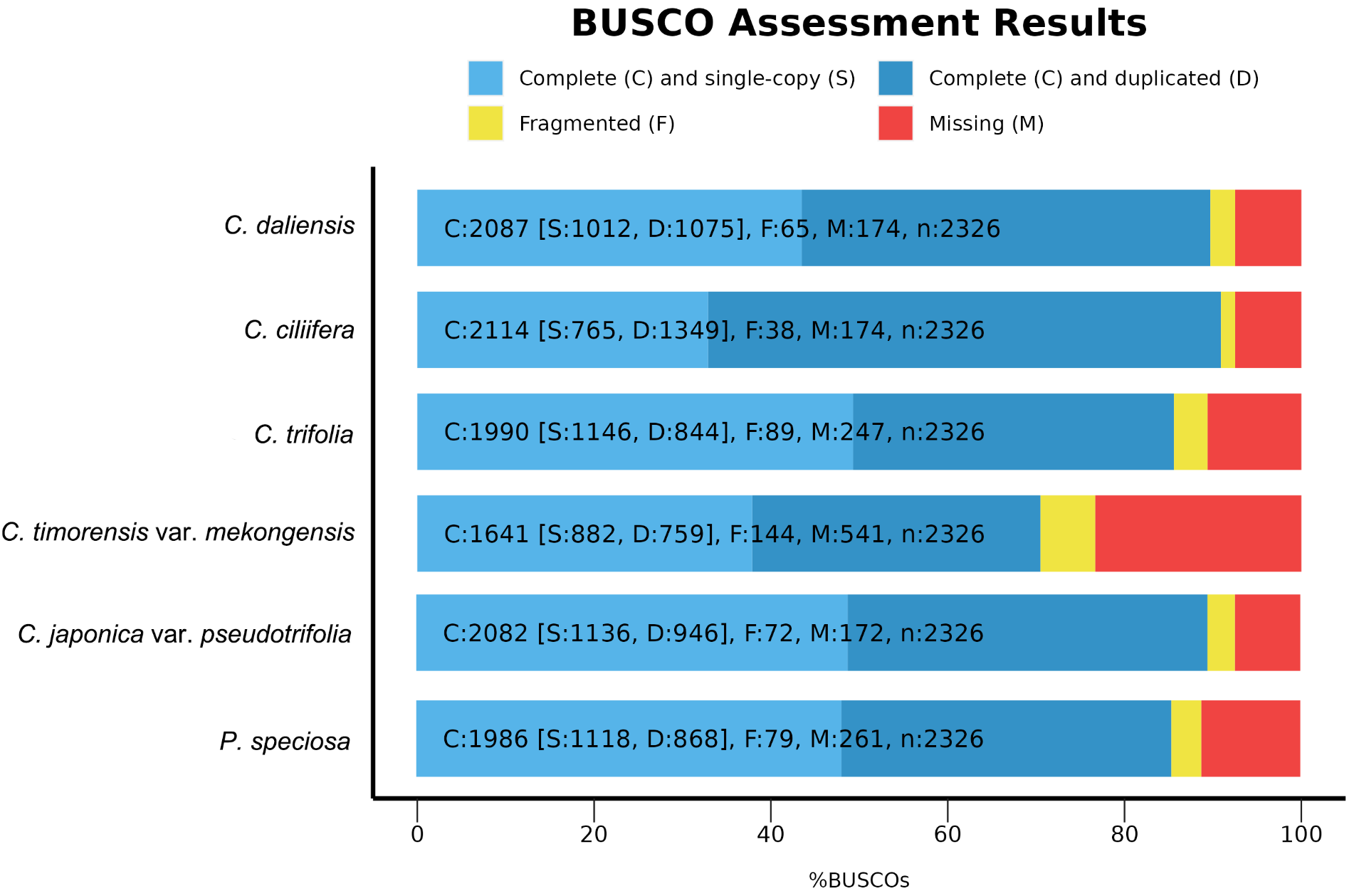


**Figure S2.** Phylogenies of *Causonis* generated with the 136taxa-810nu dataset using the maximum likelihood (ML) and Bayesian inference (BI) methods. Numbers above the branches show the ML bootstrap support/BI posterior probability values, and “*” denotes the maximal support. The main clades of *Causonis* are marked in different colors, consistent with Fig. 1.


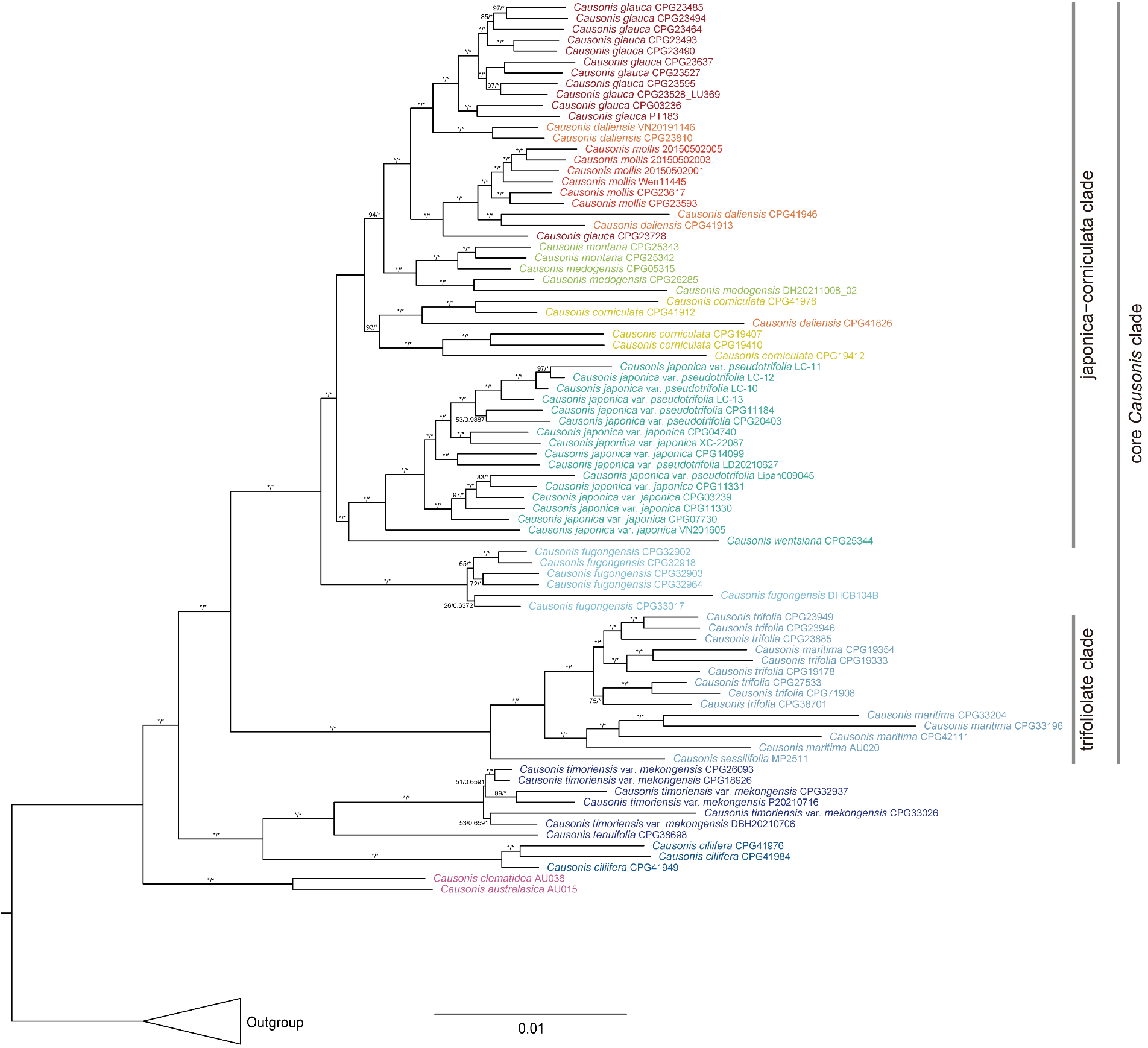


**Figure S3.** Phylogenies of *Causonis* constructed using multispecies coalescent method with (a) 136taxa-79pd dataset and (b) 136taxa-810nu dataset, showing cyto-nuclear discordance. Numbers on the branch show local posterior probability support value, and “*” denotes the maximal support. Phylogram overview of each phylogeny is shown aside. The main clades of *Causonis* are marked in different colors, consistent with Fig. 1.


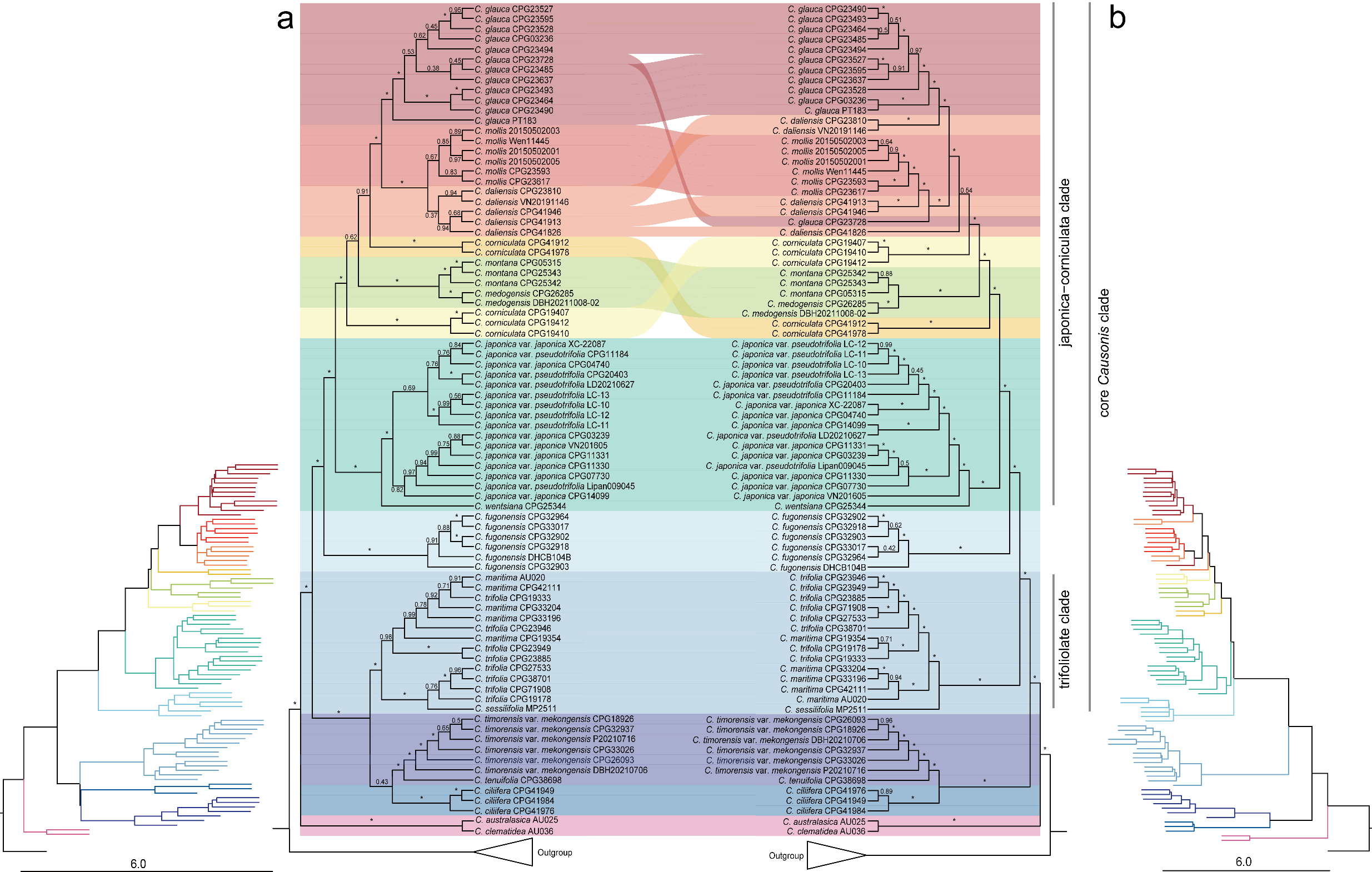


**Figure S4.** Relative importance of incomplete lineage sorting (ILS), gene tree estimation error (Est.error), and gene flow in generating gene tree variation. The percentages were estimated based on (a) LMG, (b) Last, (c) First, and (d) Pratt methods, respectively. Bars represent 95% confidence intervals. The total R^2^ = 75.6% and the metrics were normalized to sum 100%.


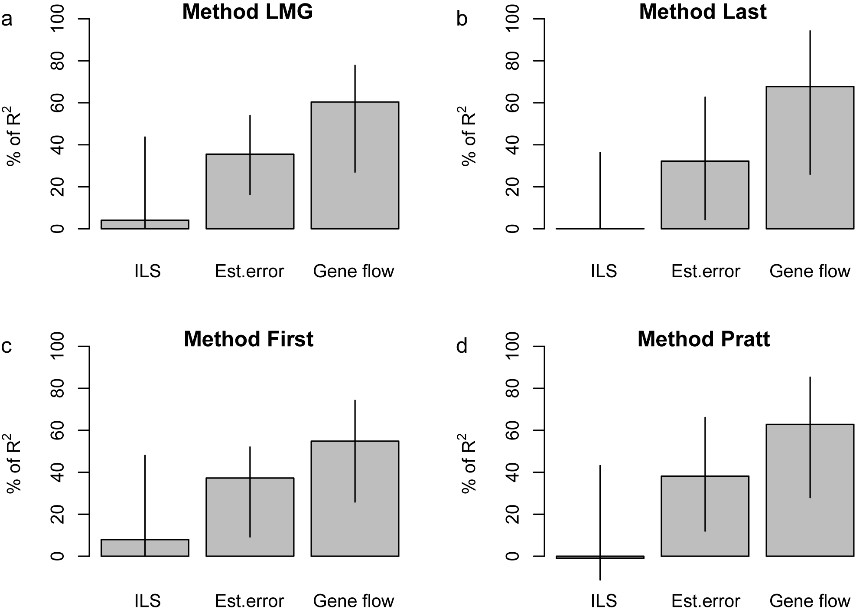


**Figure S5.** Heatmap of *D*‐statistics in the context of phylogenetic relationships for species of *Causonis*, showing introgression events between species. The color key shown on the top right indicates the values of *D*‐statistics, and the significance of the results. Phylogeny constructed with the 23taxa-810nu dataset using multispecies coalescent method is shown aside the heatmap.


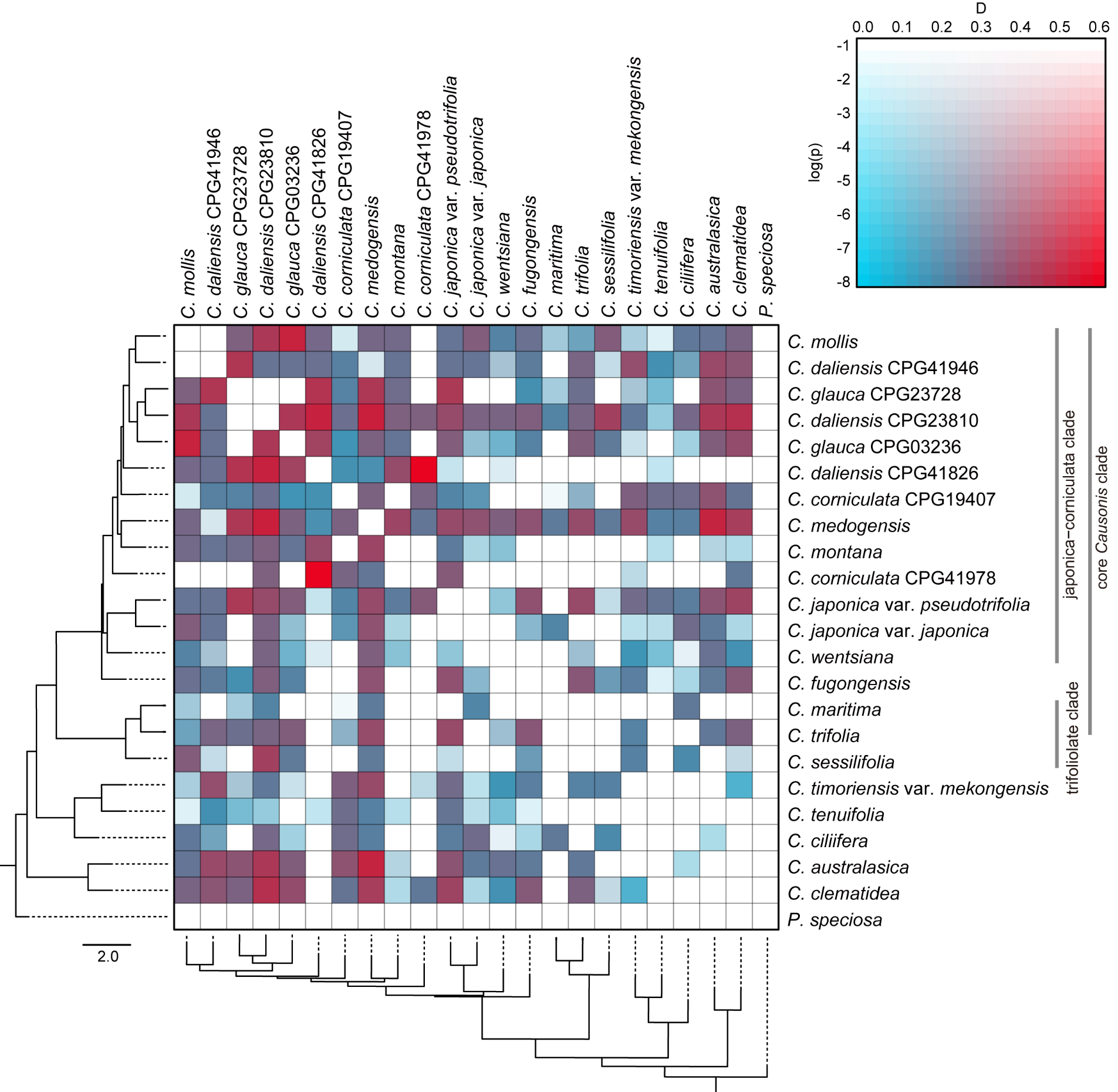


**Figure S6.** Negative log pseudolikelihood (–logplik) score profiles obtained by SNaQ in PhyloNetworks for eight species, representing the major clades of *Causonis* and the outgroup, with hmax from 0 to 5.


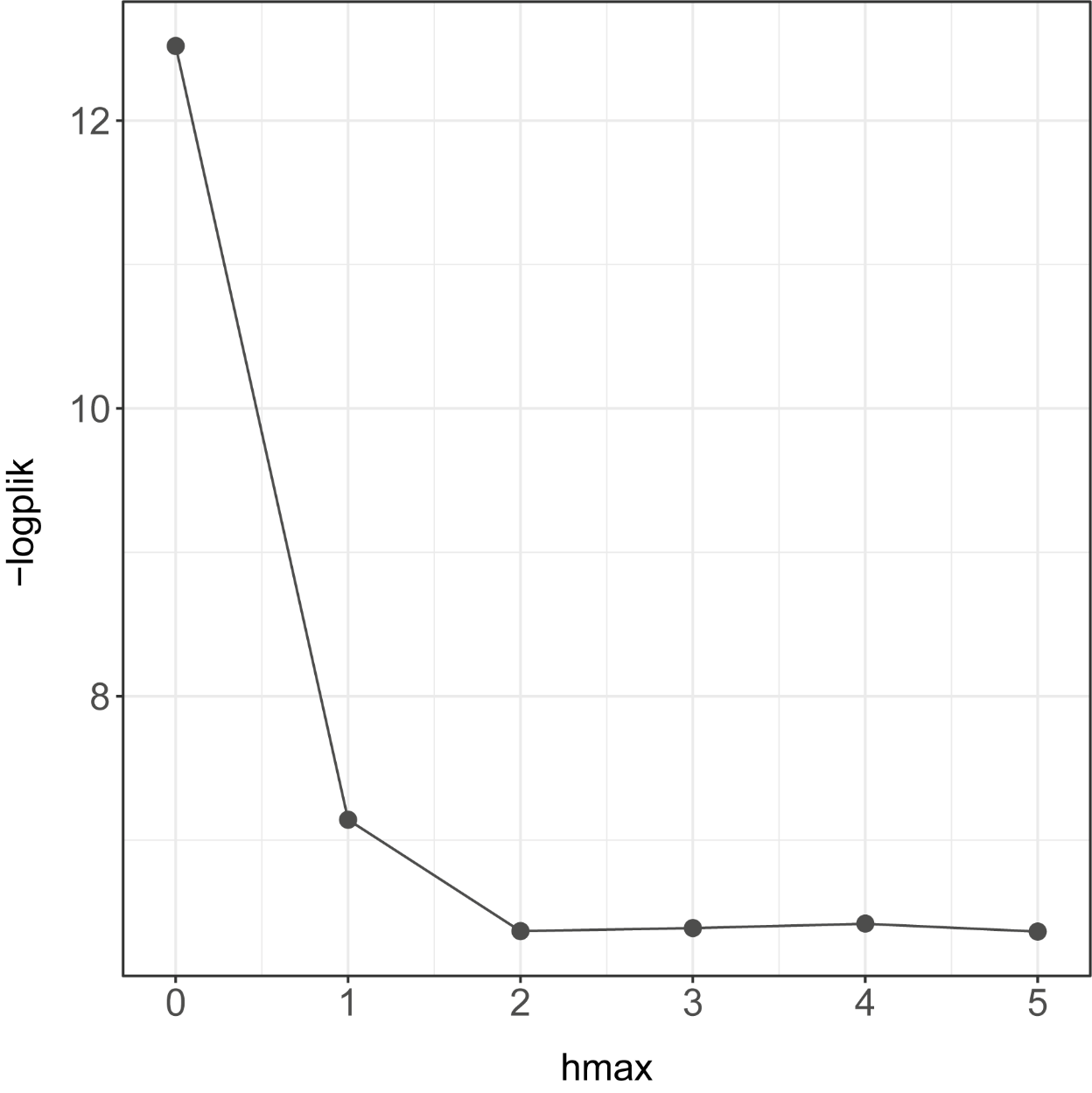


**Figure S7.** Distribution density of *Ks* distances from 0 to 3 for five species of *Causonis* and one species of *Pseudocayratia*. Colored lines denote the components inferred using a mixture model, where the red lines show an ancestral whole genome triplication event in the core eudicots.


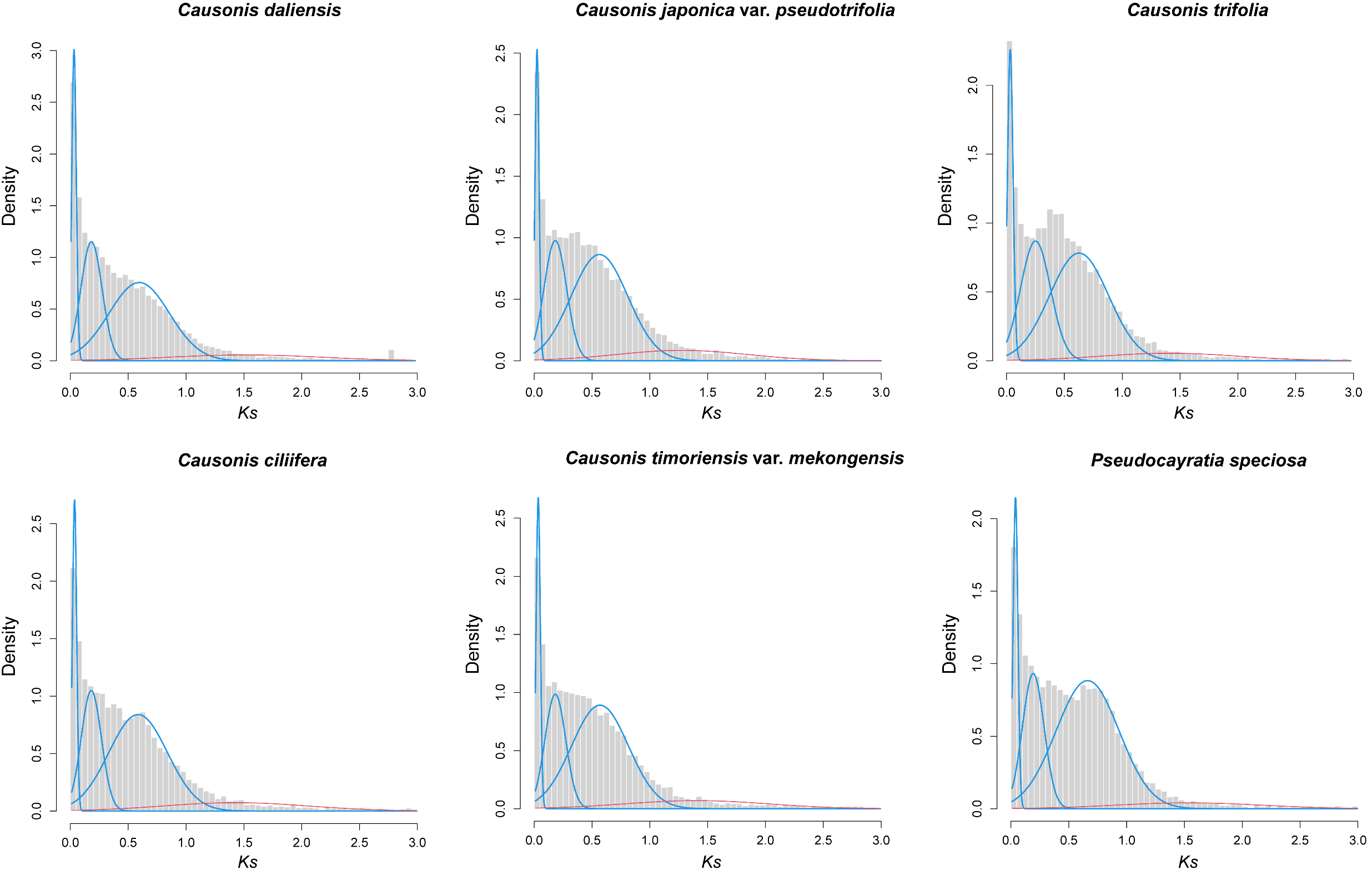


**Figure S8.** Distribution density of *Ks* distances of ortholog pairs between species pairs of *Causonis* and the distribution density of *Ks* distances of paralog pairs within the corresponding species.


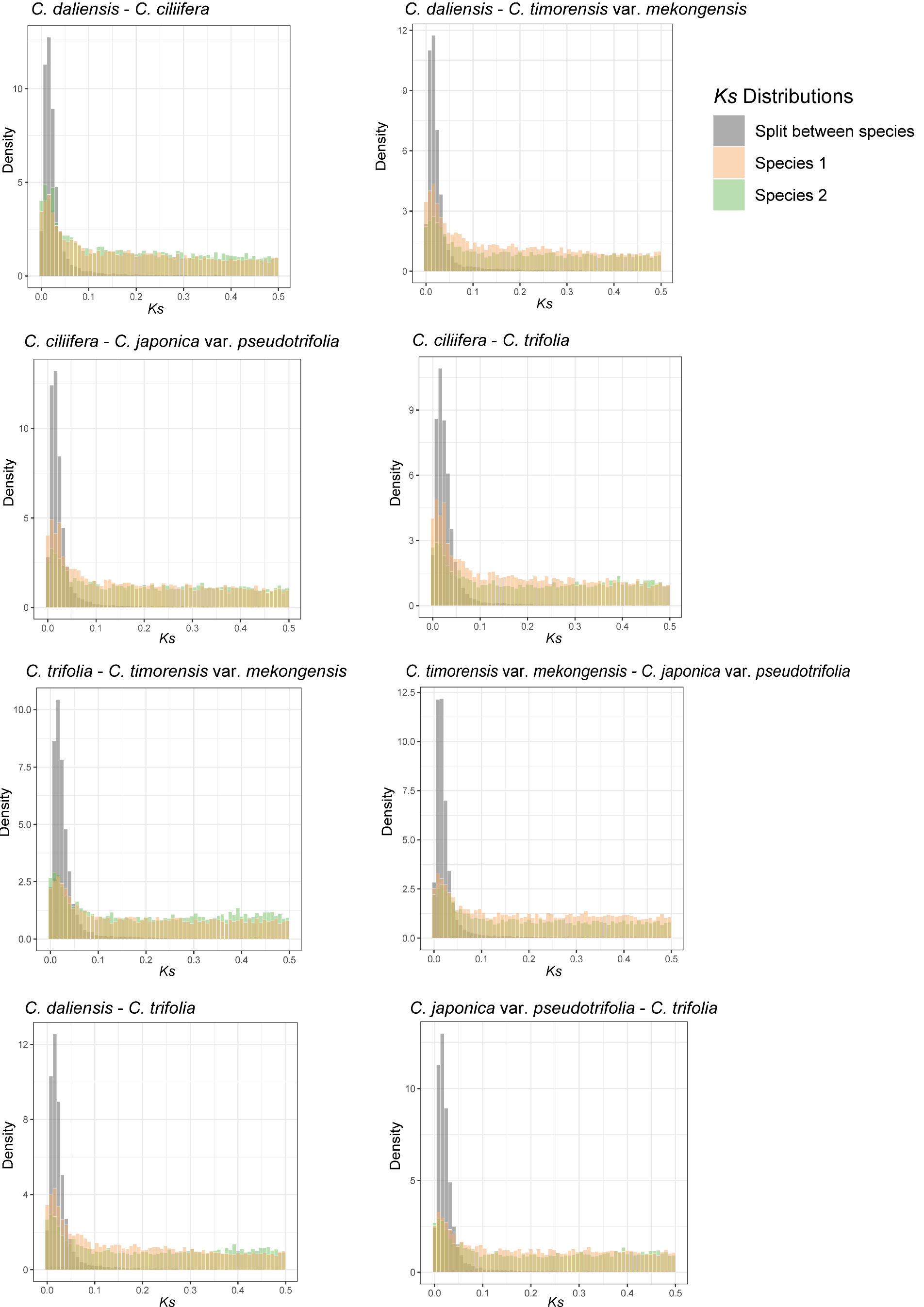


**Figure S9** The inferred gene duplication events by mapping homologous gene tree to multispecies coalescent tree using least common ancestor reconciliation. For each node, the yellow box shows the number of duplicated gene families/total gene families, and the corresponding cyan box shows the percentage of duplicated gene families. Numbers above (navy blue) and below (red) the branches denote the expansion and contraction of gene families, respectively.


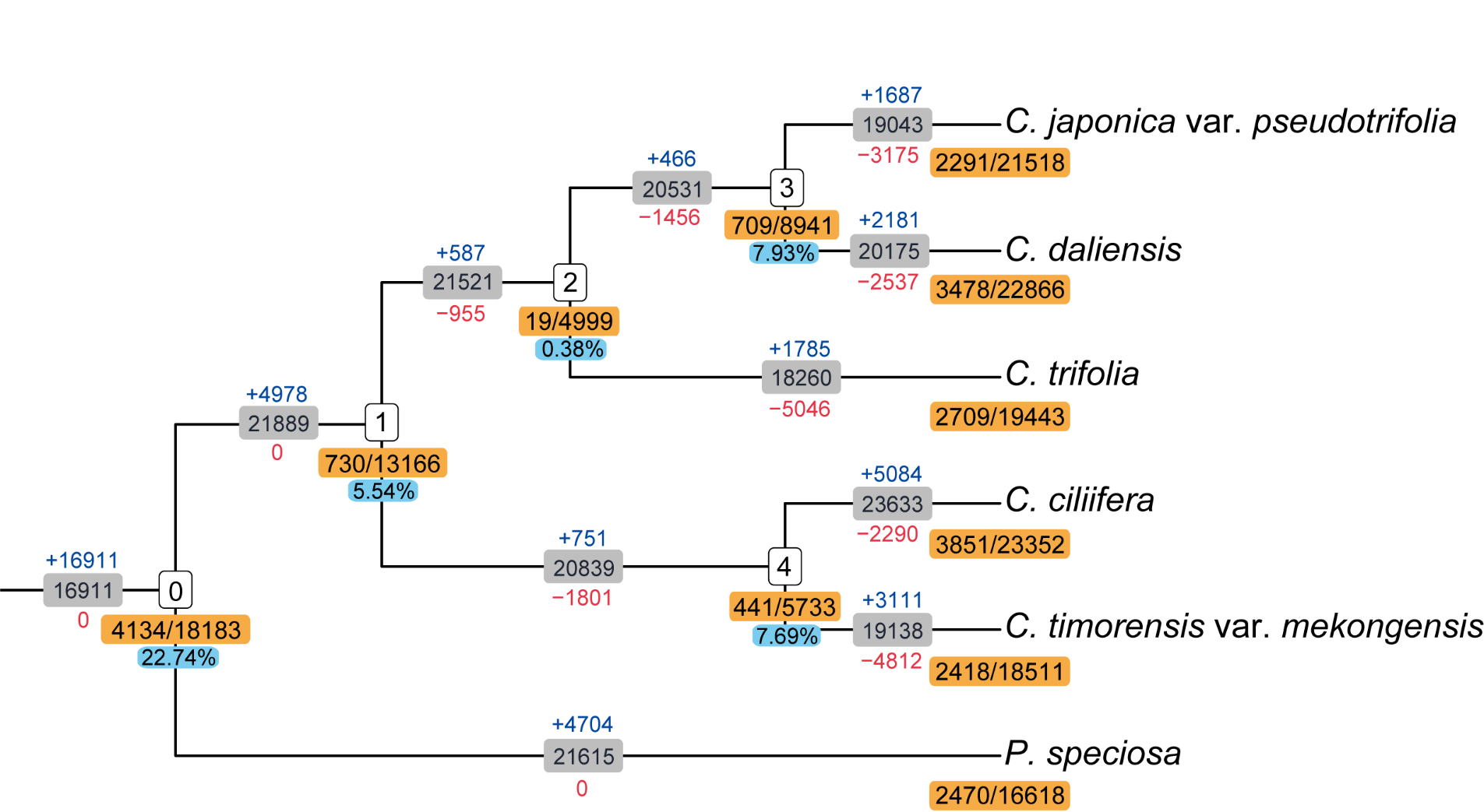


**Figure S10.** Enriched GO categories of duplicated genes derived from the allopolyploidization in at least two species of the core *Causonis*, with the genome of *Arabidopsis thaliana* as a reference. Sizes of the circles denote the proportion of genes of the indicated category in all duplicated genes (gene ratio), and the color denotes the significance level.


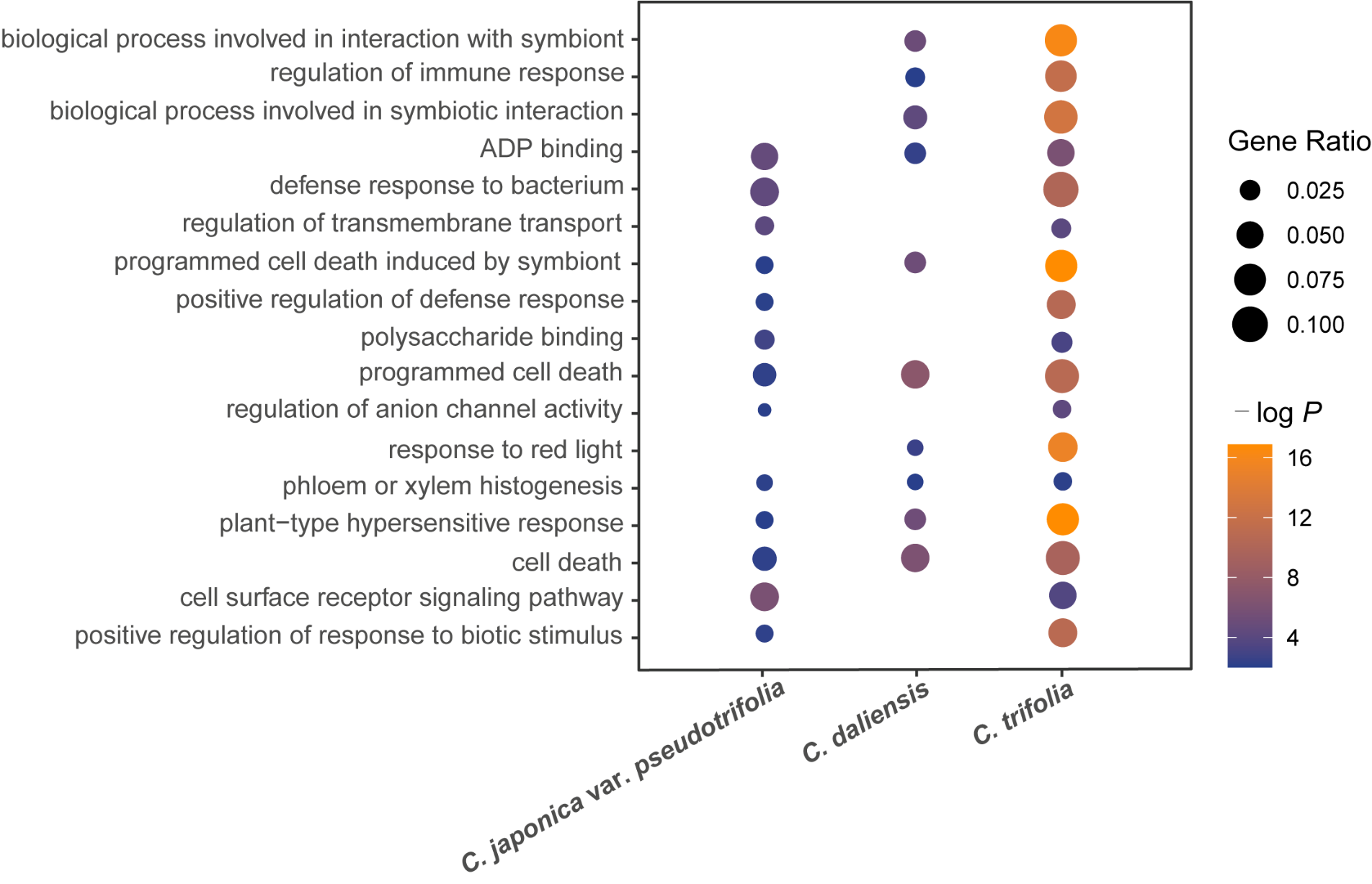


**Figure S11.** Chronogram of *Causonis* inferred from MCMCTree in PAML package based on the 76taxa-50nu dataset. The posterior distribution for each node was plotted to represent the 95% highest posterior density confidence interval of divergence time. Calibration points are indicated with stars. The estimated mean divergence times for branches within *Causonis* and three calibration points are displayed at the corresponding nodes. Ma, million years ago.


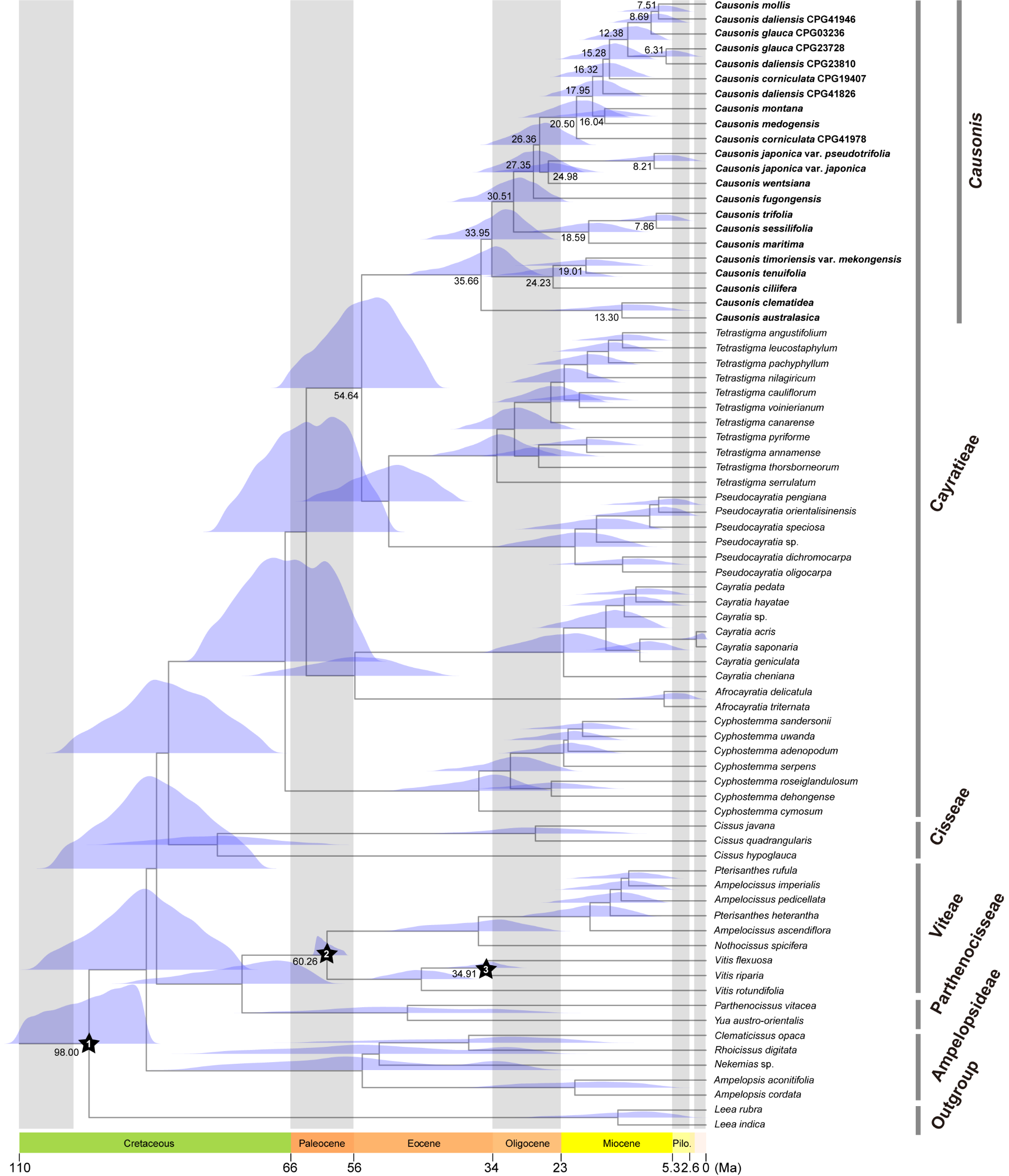


**Figure S12.** Chronogram of *Causonis* inferred from MCMCTree in PAML package based on the 76taxa-79pd dataset. Posterior distributions for each node were plotted to represent the 95% highest posterior density confidence interval of divergence times. Calibration points are indicated with stars. The estimated mean divergence time for branches within *Causonis* and three calibration points are displayed at the corresponding nodes. Ma, million years ago.


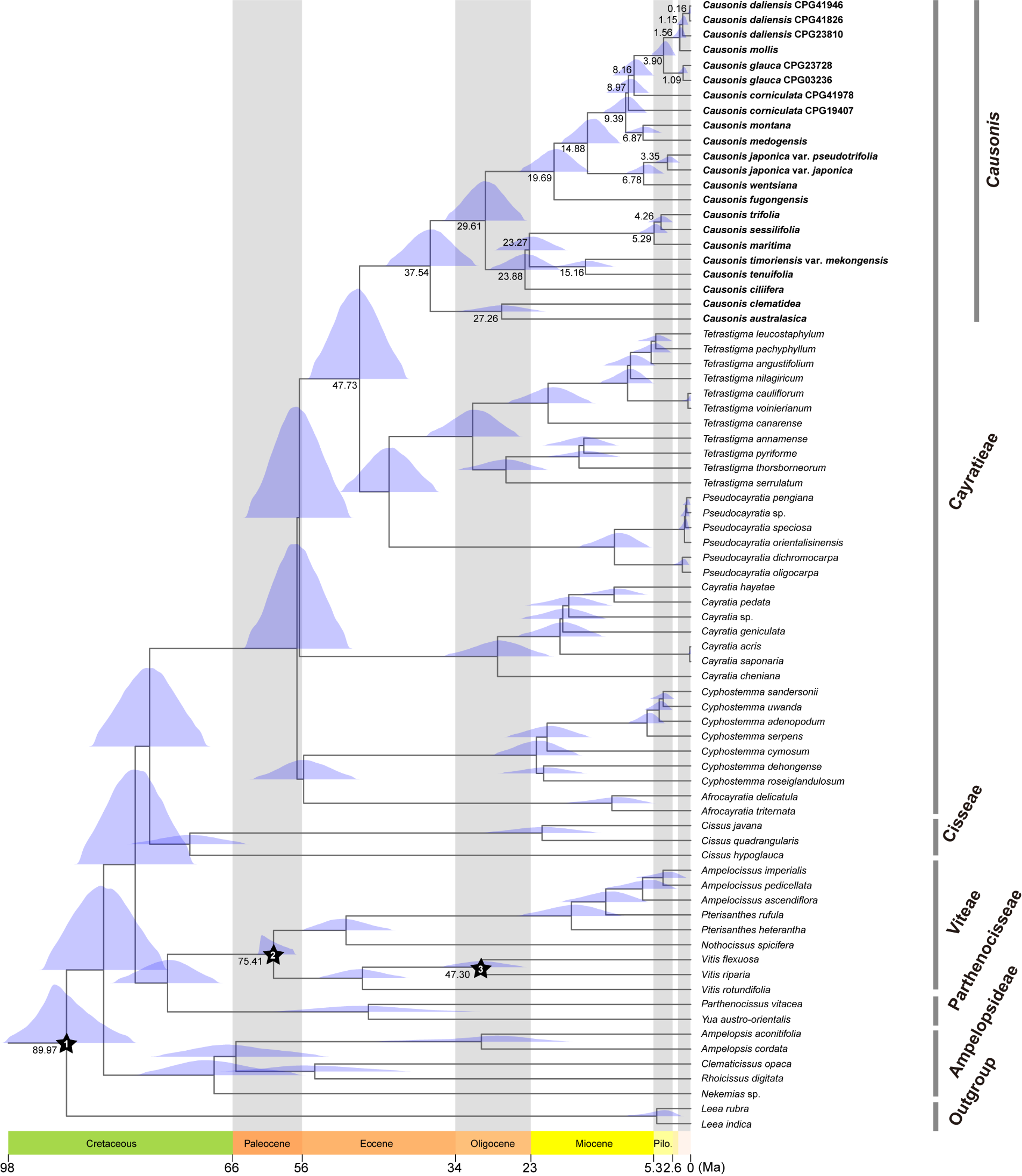


**Figure S13.** Ancestral area reconstructions for *Causonis* with BioGeoBEARS using the chronogram based on the 76taxa-79pd dataset (for concision, outgroup except for *Tetrastigma* and *Pseudocayratia* are not shown). Mean node ages are displayed at the corresponding nodes, and posterior distributions were plotted to represent the 95% highest posterior density confidence interval of divergence times. The pie charts indicate the relative possibilities of ancestral areas estimated. A, East Asia (including the East Asian monsoon region, Sino-Himalaya, and Indochina); B, the Indian subcontinent; C, the Sunda region (including the Malesian region except for New Guinea); and D, the Sahul region (including the continental Australian and New Guinea). Ma, million years ago.


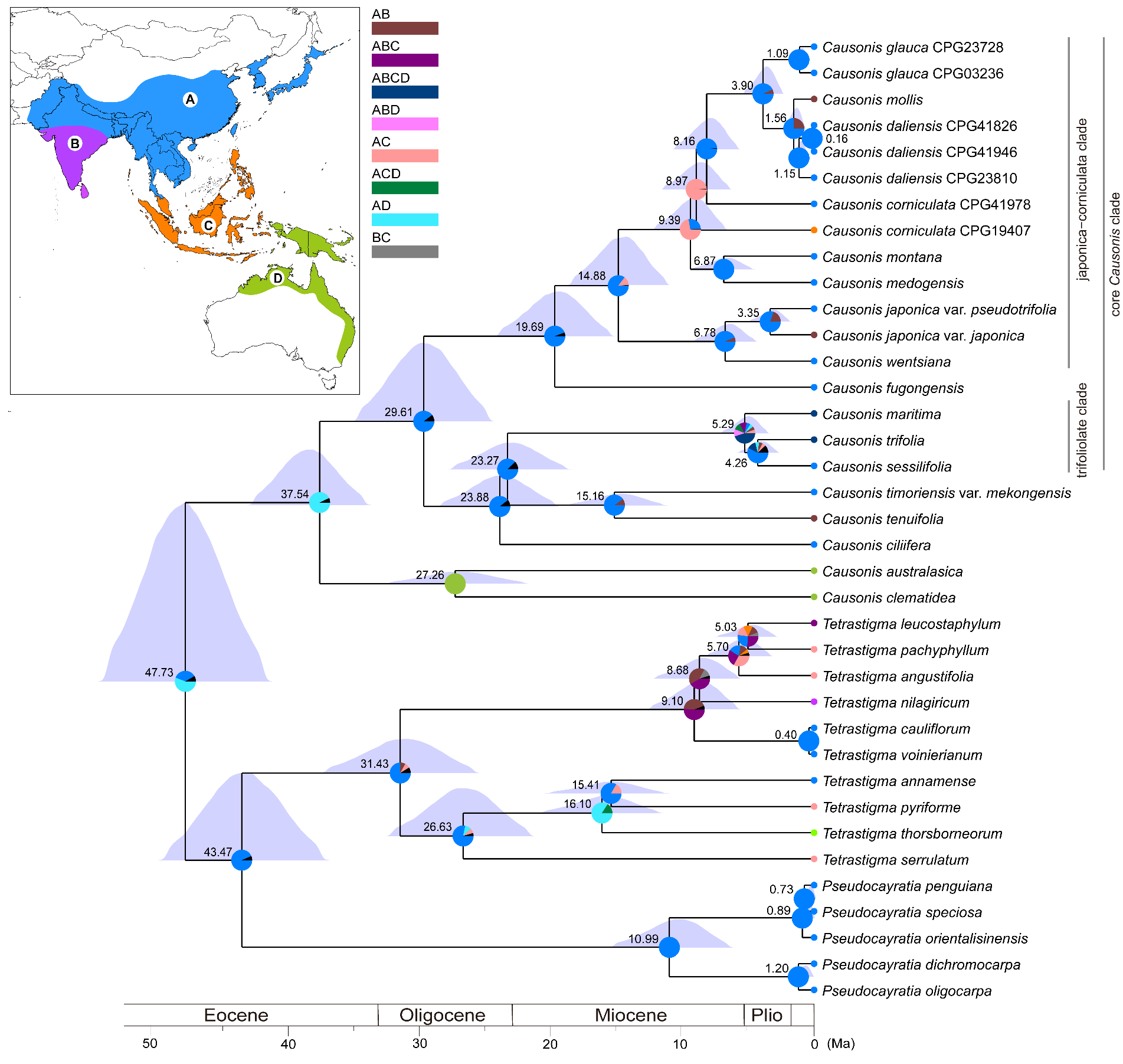

Supplement: Supplementary file 2 — Additional file 2: Fig. S1. BUSCO assessment results for the transcriptomes of five Causonis species and one Pseudocayratia species. Fig. S2. Phylogenies of Causonis generated with the 136taxa-810nu dataset using the maximum likelihood and Bayesian inference methods. Fig. S3. Phylogenies of Causonis constructed using multispecies coalescent method with 136taxa-79pd dataset and 136taxa-810nu dataset, showing cyto-nuclear discordance. Fig. S4. Relative importance of incomplete lineage sorting, gene tree estimation error, and gene flow in generating gene tree variation. Fig. S5. Heatmap of D‐statistics in the context of phylogenetic relationships for species of Causonis, showing introgression events between species. Fig. S6. Negative log pseudolikelihood score profiles obtained by SNaQ in PhyloNetworks for eight species. Fig. S7. Distribution density of Ks distances from 0 to 3 for five species of Causonis and one species of Pseudocayratia. Fig. S8. Distribution density of Ks distances of ortholog pairs between species pairs of Causonis and the distribution density of Ks distances of paralog pairs within the corresponding species. Fig. S9. The inferred gene duplication events by mapping homologous gene tree to multispecies coalescent tree using least common ancestor reconciliation. Fig. S10. Enriched GO categories of duplicated genes derived from the allopolyploidization in at least two species of the core Causonis. Fig. S11. Chronogram of Causonis inferred from MCMCTree in PAML package based on the 76taxa-50nu dataset. Fig. S12. Chronogram of Causonis inferred from MCMCTree in PAML package based on the 76taxa-79pd dataset. Fig. S13. Ancestral area reconstructions for Causonis with BioGeoBEARS using the chronogram based on the 76taxa-79pd dataset. [file 12915_2023_1718_MOESM2_ESM.docx]
